# Analysis and Optimization of Conditions for the Use of 2′,7′-Dichlorofluorescein Diacetate in Cultured Hepatocytes

**DOI:** 10.3390/antiox10050674

**Published:** 2021-04-26

**Authors:** Megan J. Reiniers, Lianne R. de Haan, Laurens F. Reeskamp, Mans Broekgaarden, Rowan F. van Golen, Michal Heger

**Affiliations:** 1 Jiaxing Key Laboratory for Photonanomedicine and Experimental Therapeutics, Department of Pharmaceutics, College of Medicine, Jiaxing University, Jiaxing 314001, China; megan.reiniers@gmail.com (M.J.R.); dehaan.lianne@gmail.com (L.R.d.H.); 2 Department of Surgery, Haaglanden Medisch Centrum, 2512 VA The Hague, The Netherlands; 3 Laboratory of Experimental Oncology, Department of Pathology, Erasmus MC, 3015 GD Rotterdam, The Netherlands; 4 Department of Vascular Medicine, Amsterdam UMC, Location AMC, 1105 AZ Amsterdam, The Netherlands; l.f.reeskamp@amsterdamumc.nl; 5 Team Cancer Targets and Experimental Therapeutics, Department Microenvironment Cell Plasticity and Signaling, Institute for Advanced Biosciences, CNRS UMR 5309, Université de Grenoble-Alpes, Allée des Alpes, 38700 La Tronche, France; mans.broekgaarden@univ-grenoble-alpes.fr; 6 INSERM U 1209, Allée des Alpes, 38700 La Tronche, France; 7 Department of Gastroenterology and Hepatology, Leiden University Medical Center, 2333 ZA Leiden, The Netherlands; R.F.van_Golen@lumc.nl; 8 Department of Pharmaceutics, Utrecht Institute for Pharmaceutical Sciences, Utrecht University, 3584 CG Utrecht, The Netherlands

**Keywords:** fluorogenic redox probe, oxidative and nitrosative stress, liver diseases, hepatocytes, cellular uptake and export, intravital fluorescence imaging, anoxia and reoxygenation

## Abstract

Numerous liver pathologies encompass oxidative stress as molecular basis of disease. The use of 2′,7′-dichlorodihydrofluorescein-diacetate (DCFH_2_-DA) as fluorogenic redox probe is problematic in liver cell lines because of membrane transport proteins that interfere with probe kinetics, among other reasons. The properties of DCFH_2_-DA were analyzed in hepatocytes (HepG2, HepaRG) to characterize methodological issues that could hamper data interpretation and falsely skew conclusions. Experiments were focused on probe stability in relevant media, cellular probe uptake/retention/excretion, and basal oxidant formation and metabolism. DCFH_2_-DA was used under optimized experimental conditions to intravitally visualize and quantify oxidative stress in real-time in HepG2 cells subjected to anoxia/reoxygenation. The most important findings were that: (1) the non-fluorescent DCFH_2_-DA and the fluorescent DCF are rapidly taken up by hepatocytes, (2) DCF is poorly retained in hepatocytes, and (3) DCFH_2_ oxidation kinetics are cell type-specific. Furthermore, (4) DCF fluorescence intensity was pH-dependent at pH < 7 and (5) the stability of DCFH_2_-DA in cell culture medium relied on medium composition. The use of DCFH_2_-DA to measure oxidative stress in cultured hepatocytes comes with methodological and technical challenges, which were characterized and solved. Optimized in vitro and intravital imaging protocols were formulated to help researchers conduct proper experiments and draw robust conclusions.

## 1. Introduction

Oxidants in the form of reactive oxygen and nitrogen species (ROS and RNS, respectively), redox-active transition metals such as Fe^2+^ and Cu^+^, as well as activated peroxidases (e.g., cytochrome c peroxidase) are chemically reactive compounds that are able to (ir)reversibly alter the structure of (bio)molecules [1]. ROS/RNS are generated intracellularly by enzymatic sources (e.g., cytochrome P450 enzymes [2]) as well as non-enzymatic sources (e.g., oxidative phosphorylation in mitochondria [3]) and are essentially involved in cell signaling pathways when formed under controlled conditions. Transition metals are generally kept in a protein-bound state to control their reactivity, although a labile pool of free Fe^2+^ and Cu^+^ exists intracellularly and is maintained within tight concentration limits under non-pathological circumstances [4]. Under pathological circumstances, however, oxidant levels can rise due to the increased formation of ROS/RNS, augmentation of the labile Fe^2+^/Cu^+^ pool, and/or a reduction in the (extra)cellular antioxidative capacity. A state of oxidative stress consequently ensues [1] that has been causally related to, e.g., cardiovascular-, neurodegenerative-, malignant-, and liver diseases [5]. Hepatocytes are particularly prone to developing oxidative stress because of their large number of mitochondria and ROS/RNS-producing enzymes, as well as their principal role in copper and iron metabolism [1]. Accordingly, oxidative stress contributes to many forms of liver disease [1,6] and therefore constitutes a major research topic within the field of hepatology.

**Figure 1:**
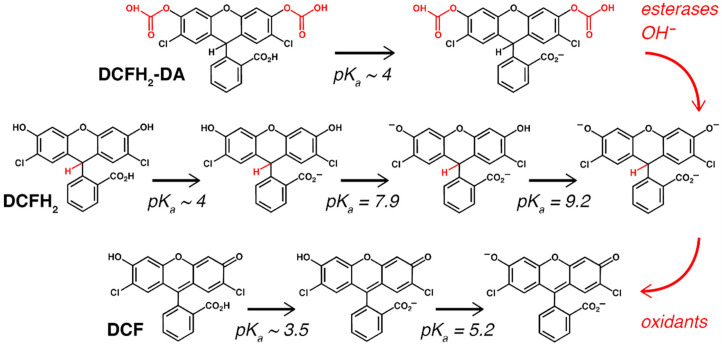
The chemical structure, pH-dependent isoforms, and (estimated) acid dissociation constants (pK_a_) [14,15] of DCFH_2_-DA, DCFH_2_, and DCF. Details are provided in the text.

Because of their high reactivity and short half-lives oxidants are difficult to measure directly, particularly under in vitro conditions. Consequently, fluorogenic and luminogenic redox probes have emerged over the past decades as the preferred tool to measure oxidative stress in vitro due to their easy use, low cost, and non-toxicity [7,8]. Of the different probes available, the fluorogenic 2′,7′-dichlorodihydrofluorescein-diacetate (DCFH_2_-DA) is amongst the most frequently used redox probes. The acetate groups on DCFH_2_-DA allow for diffusion across the plasma membrane, after which both groups are cleaved by intracellular esterases to form DCFH_2_ that is believed to be retained in the cytosol (Figure 1). DCFH_2_ is reactive towards many types of oxidants, including nitrogen dioxide (^•^NO_2_) [9], the carbonate radical anion (CO_3_^•−^) [9], the hydroxyl radical (^•^OH) [9], Fe^2+^ [10], Cu^+^ [10], thiyl radicals (e.g., the glutathione radical; GS^•^) [11], and peroxidases (e.g., cytochrome c peroxidase) [12]. Following two-electron oxidation, in which superoxide anion (O_2_^•^^−^) is generated as by-product [13], fluorescent DCF is formed that can be visualized or quantified as a nonspecific measure of oxidative stress (Figure 1).

Despite its widespread application, only limited research has focused on the practical aspects of the in vitro use of DCFH_2_-DA [16,17,18,19,20,21,22,23,24]. More specifically, studies on DCFH_2_-DA uptake, DCFH_2_ oxidation, and DCF retention in liver cell lines or hepatocytes are lacking. The probe kinetics in vitro are of particular concern given that hepatocytes express a multitude of membrane transporters that have important practical implications for the use of DCFH_2_-DA [25]. Inasmuch as primary hepatocytes swiftly de-differentiate in monolayer culture, hepatoma cell lines are commonly employed to study hepatic (patho)physiology in vitro. HepG2 is a human hepatocellular carcinoma-derived cell line that is one of the most widely used cell types for these purposes. The relatively new HepaRG cell line is an increasingly used human hepatocellular carcinoma-derived cell line that has the ability to differentiate over a 4-wk period into a heterogeneous monolayer culture consisting of islets of hepatocyte-like cells that are surrounded by cholangiocyte-like cells [26].

In light of the abovementioned knowledge gaps, this study aimed to optimize the practical applicability of DCFH_2_-DA in HepG2 cells and undifferentiated (2-wk old) as well as differentiated (4-wk old) HepaRG cells. The most important observations were that extracellular DCF enters HepG2 and HepaRG cells, that DCF is poorly retained in hepatocytes, and that DCFH_2_ oxidation kinetics in hepatocytes are cell type-specific. Moreover, DCF fluorescence intensity was pH-dependent at pH < 7 and the stability of DCFH_2_-DA in cell culture medium relied on medium composition. A detailed experimental protocol that corrects for these technical hurdles was developed with the intent to aid researchers in optimizing experimental design and proper analysis of data generated with DCFH_2_-DA. To further demonstrate the probe’s utility under these experimental conditions, DCFH_2_-DA was used to visualize and quantify oxidative stress in real-time in HepG2 cells subjected to anoxia/reoxygenation.

## 2. Methods

References to online supplemental material are indicated with prefix ‘S.’

### 2.1. Reagents and Buffers

DCFH_2_-DA was purchased from Life Technologies/Molecular Probes (Eugene, OR, USA) and dissolved in methanol (MeOH) at a 25-mM stock concentration or in dimethylsulfoxide (DMSO) at a 50-mM stock concentration. DCF was acquired from Sigma-Aldrich (St. Louis, MO, USA) and dissolved in DMSO at a 20-mM stock concentration. All other reagents are listed in Table S1. The concentrations listed throughout this manuscript refer to final concentrations unless indicated otherwise.

### 2.2. Preparation of DCFH_2_

High-purity DCFH_2_ was prepared from DCFH_2_-DA in accordance with an optimized and validated protocol [10]. Briefly, 5 μmol of DCFH_2_-DA in MeOH was dissolved 2.5 mL of 100 mM NaOH and incubated for 15 min at room temperature (RT) in the dark to ensure complete deacetylation of DCFH_2_-DA to DCFH_2_. The solution was subsequently adjusted to pH = 1 by the addition of 2.5 mL of 200 mM HCl to precipitate DCFH_2_. Next, liquid phase extraction of DCFH_2_ was performed by the addition of 4 mL of chloroform (CHCl_3_) to the suspension. After vortexing, the organic phase was aspirated and evaporated under a stream of N_2_ gas at RT in the dark. Subsequently, the crystallized DCFH_2_ was dissolved in MeOH to yield a 92-mM stock solution and stored under N_2_ gas at −20 °C.

### 2.3. Determination of Molar Extinction Coefficients

The molar extinction coefficient (ε) of DCFH_2_-DA was determined in water, HEPES buffer (10 mM HEPES, 0.88% NaCl, pH = 7.4, 0.292 osmol/kg), and MeOH. DCFH_2_-DA in DMSO was diluted in the solvent of interest (0–20 μM) and sample absorbance was determined at 258, 259, or 260 nm for water, HEPES buffer, or MeOH, respectively, by UV/VIS absorption spectroscopy (Lambda 18, Perkin Elmer, Waltham, MA, USA) in a 1-cm path length quartz cuvette (Hellma Analytics, Müllheim, Germany). The molar extinction coefficient was subsequently calculated over the complete concentration range in all solvents according to the Beer-Lambert equation [27].

The molar extinction coefficient of DCFH_2_ in MeOH was recently reported [10]. In addition, the molar extinction coefficient of DCFH_2_ was determined in water, PBS (pH = 6 or pH = 12), and HEPES buffer (adjusted to pH = 6 or pH = 12 with HCl or NaOH, respectively). DCFH_2_ in MeOH was prepared in the solvent of interest (0–90 μM) and sample absorbance was determined at 286 nm for water, PBS (pH = 6), and HEPES buffer (pH = 6), and at 305 nm for PBS and HEPES buffer (both pH = 12).

Similarly, DCF in DMSO was diluted in water (pH = 12), HEPES buffer, MeOH, and DMSO (0–20 μM) and its molar extinction coefficient was calculated at 503 nm, 284 nm, and 535 nm for aqueous solvent (water and HEPES buffer), MeOH, and DMSO, respectively.

### 2.4. Spectral Properties of DCFH_2_-DA, DCFH_2_, and DCF

Concentration-dependent (0–20 μM) ground state absorption spectra were recorded for DCFH_2_-DA, DCFH_2_, and DCF in aqueous (MilliQ, HEPES buffer) and organic solvents (MeOH, DMSO).

In addition, pH-dependent absorption spectra of 20 μM DCF and DCFH_2_ as well as pH-dependent excitation and emission spectra (Cary Eclipse, Varian, Palo Alto, CA, USA) of 20 μM DCF were acquired in unbuffered water adjusted to pH = 1–12 with 37% HCl.

### 2.5. Stability of DCFH_2_-DA and DCFH_2_ in Solvent

The stability of DCFH_2_-DA and DCFH_2_ was determined in organic and aqueous solvents. The stability of DCFH_2_ and DCFH_2_-DA in MeOH is reported elsewhere [10]. A 20-μM solution of each compound was prepared in DMSO, HEPES buffer, and water. Samples were stored at −20 °C or 4 °C in the dark and the extent of auto-oxidation (i.e., the formation of DCF) was determined spectrofluorometrically (λ_ex_ = 503 ± 5 nm, λ_em_ = 513–650 nm or λ_ex_ = 535 ± 5 nm, λ_em_ = 545–650 nm for water/HEPES or DMSO, respectively) at different time points over a period of 28 d. The fluorescence emission spectra were integrated and plotted as a percentage of the integrated spectra of a freshly prepared 20-μM DCF sample (reference standard).

The short-term stability of DCFH_2_-DA was analyzed spectrofluorometrically in PBS and serum-free cell culture media (WE, DMEM, and RPMI-1640) with or without HEPES (25 mM, pH = 7.4) at ambient CO_2_ tension. The spectrofluorometer was employed in kinetics mode (λ_ex_ = 500 ± 5 nm, λ_em_ = 523 ± 5 nm) using continuous magnetic stirring and Peltier-controlled temperature regulation. Solvents/reagents were added to the cuvette in the following order: t = 0 min, 1494 μL of solvent (equilibrated at 37 °C) and t = 1 min, 6 μL of 5 mM DCFH_2_-DA in DMSO (20 μM final concentration). Fluorescence was recorded over a period of 2 h with continuous acquisition. The fluorescence emission intensity at each time point was plotted as a percentage of the fluorescence emission intensity of a 20-μM DCF reference sample, measured at the end of the kinetics read.

### 2.6. Cell Culture

HepaRG cells were kindly provided by Biopredic International (Saint-Grégoire, France). HepG2 cells were purchased from ATCC (Manassas, VA, USA). Both cell lines were cultured in WE medium supplemented with 10% (vol/vol) fetal bovine serum, 100 U/mL penicillin, 100 μg/mL streptomycin, 2 mM L-glutamine, 5 μg/mL insulin, and 50 mM hydrocortisone under standard culture conditions (humidified atmosphere of 95% air and 5% CO_2_ at 37 °C). Cells were subcultured at a 1:5 ratio (HepG2) or 1:7 ratio (HepaRG) following detachment by trypsinization (15 min at 37 °C) in a 2:1:1 accutase:accumax:PBS mixture. HepG2 cells, undifferentiated HepaRG cells, and differentiated HepaRG cells were used for experiments after 4–5, 12–16, and 26–30 d of culture, respectively. Cells were seeded in 24-wells plates and used at 100% confluence for each experiment.

### 2.7. Cellular DCFH_2_-DA Uptake

Time- and concentration-dependent DCFH_2_-DA uptake were analyzed by incubating cells with 50 μM DCFH_2_-DA in PBS for 0–20 min or with 0–100 μM DCFH_2_-DA in PBS for 15 min, respectively. Following incubation, the DCFH_2_-DA-containing PBS was aspirated, snap frozen in liquid N_2_, and stored at −20 °C. At a later time point, samples were thawed and centrifuged for 15 min at 15,000× *g* (4 °C) to pellet any cellular debris. Next, 400 μL supernatant was incubated with 600 μL of 190 mM NaOH for 15 min at RT in the dark so as to convert all DCFH_2_-DA into DCFH_2_ [10]. The concentration of DCFH_2_ and DCF (from concurrent auto-oxidation) was determined in each sample by means of absorbance (ε_305 nm_ = 7906 M^−1^ · cm ^−1^ in PBS, pH = 12) and fluorescence spectroscopy (λ_ex_ = 503 ± 5 nm, λ_em_ = 513–700 nm against a 0–40-nM DCF standard curve), respectively. The cellular uptake of DCFH_2_-DA was calculated by subtracting the combined nanomolar amount of DCFH_2_ and DCF in the supernatant (as a measure of residual DCFH_2_-DA following incubation) from that of the DCFH_2_-DA in PBS stock solution (n = 4). Data were subsequently normalized to total protein content per well that was determined in duplicate using a colorimetric commercial kit (bicinchoninic acid [BCA] protein assay, Thermo Scientific, Rockford, IL, USA), as well as to incubation time for the concentration-dependent experiments. The cell lysis solution (0.1 M NaOH and 1% Triton X-100 in water) was chosen because of the different mechanism of cell lysis by NaOH and Triton X-100 and because these components do not interfere in the BCA derivatization reaction and subsequent spectrophotometric determination (described in Section S2.7 and Figures S1 and S2).

### 2.8. Cellular DCF Uptake

To investigate time-dependent uptake of DCF, cells were incubated with 50 μM DCF in serum-free WE medium for 0–20 min under standard culture conditions. At each time point, cells were washed twice with PBS and lysed with lysis solution (250 μL/well) for 1 h at 37 °C. Following homogenization of cell lysates by rocking, DCF fluorescence was measured using a microplate reader (λ_ex_ = 460 ± 40 nm and λ_em_ = 520 ± 20 nm; BioTek Instruments, Winooski, VT, USA). Data were corrected for total protein content per well as described above and normalized to controls (i.e., 0 min incubation).

Concentration-dependent DCF uptake was determined by incubating cells with 0–100 μM DCF in serum-free WE medium for 20 min under standard culture conditions. Subsequently, cells were washed twice in PBS and 300 μL of PBS was added to each well. DCF fluorescence was measured at abovementioned settings, corrected for total protein content per well as well as for incubation time, and normalized to control (i.e., 0 μM DCF).

### 2.9. Intracellular DCF Retention and Transmembrane Diffusion

The extent of intracellular DCF retention was determined by incubating cells in serum-free WE medium with 100 μM DCF or solvent control for 2 h at standard culture conditions. Cells were washed twice in PBS and 500 μL of serum-free WE medium was added to each well, which was aspirated at various time points over a 20-min period. Next, cells were lysed and DCF fluorescence in the lysate was determined as described above.

Intracellular DCF retention was also visualized by laser-scanning confocal microscopy (Leica SP8, Leica Microsystems, Wetzlar, Germany). HepG2 cells were grown to confluence in 6-well plates pre-coated with rat tail-derived collagen I in 0.1 M acetic acid in water for 6 h (8 μg/cm^2^). Prior to imaging, cells were incubated with 100 μM DCF in serum-free WE medium for 2 h at standard culture conditions. Thereafter, cells were washed twice with PBS and fixed in 1.5 mL of fixative (4% paraformaldehyde and 2% sucrose in PBS, 5 min, RT) following 5- or 30-min incubation with serum-free WE medium. Cells were stained with 1 μM Nile Red in PBS (from 5 mM Nile Red in DMSO stock solution) for 60 s and washed thrice with 1 mL of PBS [28,29]. The coverslips were then mounted on microscope slides using DAPI-containing Vectashield mounting medium (Vector Laboratories, Burlingame, CA, USA). Fluorescence intensity was measured per fluorophore at λ_ex_ = 405 nm, λ_em_ = 415–480 nm for DAPI; λ_ex_ = 540 nm, λ_em_ = 550–650 nm for Nile Red; and λ_ex_ = 495 nm, λ_em_ = 520–580 nm for DCF. An overlay image was composed of the individually acquired images. Laser and detector settings were kept constant throughout the experiment.

The transmembrane diffusibility of DCF was assessed using liposomes encapsulating DCF or 6-carboxyfluorescein (CF) at a self-quenching concentration. CF (49 mM) and DCF (18 mM) were prepared in 98.4 and 37.3 mM NaOH in water, respectively, and incubated overnight at 37 °C under continuous shaking. Both solutions were gradually titrated to pH = 7.4 with 37% HCl under continuous magnetic stirring, after which solvent osmolarity was determined as described in [30] against a 0–154-mM NaCl in water (pH = 7.4) standard curve and adjusted to 0.292 osmol/kg with NaCl. Liposomes were prepared by the lipid film hydration technique as described in [10]. Briefly, a lipid film was prepared by mixing stock solutions of DMPC, cholesterol, lactosyl-PE, and GM1 at a 50:40:5:5 molar ratio (10 mM final lipid concentration). The solvent was evaporated under a stream of N_2_ gas followed by 30 min of vacuum desiccation. The resulting lipid film was hydrated with 500 μL of self-quenching DCF or CF solution and sonicated using a tip sonicator (Branson Ultrasonics, Danbury, CT, USA) for 5 min. Unencapsulated probe was subsequently removed by size exclusion chromatography according to [31].

The release of encapsulated DCF or CF was determined over a period of 10 min by fluorescence spectroscopy (λ_ex_ = 491 ± 5 nm, λ_em_ = 523 ± 5 nm). A cuvette containing 1500 μL of HEPES buffer was placed into the spectrometer (maintained at 22 °C) and time-based acquisition was started. At t = 1 min, 40 μL of iso-osmotic liposome solution was added. At t = 9 min, 15 μL of 15% TX-100 in water was added to lyse the liposomes, resulting in the release of all encapsulated probe and hence maximum fluorescence intensity.

### 2.10. Basal Oxidant Formation and Cellular Metabolic Rate

To analyze the intracellular basal oxidant formation, cells were incubated with 0–100 μM DCFH_2_-DA in serum-free HEPES-buffered WE medium (25 mM, pH = 7.4) at 37 °C in a microplate reader in which DCF fluorescence was measured over a period of 2 h at 10-min intervals (λ_ex_ = 460 ± 40 nm, λ_em_ = 520 ± 20 nm). A cell-free plate was analyzed directly thereafter to determine the rate of DCFH_2_-DA auto-oxidation in the incubation medium. Cellular DCF fluorescence was adjusted for DCFH_2_-DA auto-oxidation over time and normalized to total protein content per well.

Cellular O_2_ consumption and extracellular acidification rate (ΔpH) were determined as a measure for the overall metabolic rate in cells seeded onto 96-well plates (Seahorse Bioscience, North Billerica, MA, USA). Cells were analyzed in 200 μL serum- and bicarbonate-free DMEM using a Seahorse XF96 analyzer (Seahorse Bioscience; n = 24 per cell type, 3 measurements per sample). All data were normalized to total protein content per well.

### 2.11. Real-Time Analysis of Oxidant Formation during In Vitro Anoxia/Reoxygenation in HepG2 Cells

DCFH_2_-DA was used to visualize acute oxidative stress in HepG2 cells under experimental conditions emulating hepatic ischemia-reperfusion [32]. For this purpose, a custom-built fluorescence microscopy-based experimental setup was used (Figure S3). HepG2 cells were cultured on collagen-coated (as described above) 0.8-mm channel slides (Ibidi, Planegg, Germany).

During the experiment, cells were perfused (80 μL/min) with serum-, glucose-, and pyruvate-free WE medium for 4 h at 37 °C. The medium was continuously purged with a mixture of 95% N_2_ and 5% CO_2_ or 95% air and 5% CO_2_ (Linde Gas Benelux, Schiedam, The Netherlands) for the anoxia-reoxygenation (A/R) or control group, respectively. The slide was subsequently perfused with 100 μM DCFH_2_-DA in the respective incubation medium for 15 min (80 μL/min). In an additional A/R intervention group, 1 mM dimethyl malonate (DMM), which prevents the build-up of succinate during anoxia and hence reduces oxidant formation upon reoxygenation [33], was added to the perfusion medium and the DCFH_2_-DA-containing incubation medium.

Following incubation with DCFH_2_-DA, perfusion was resumed with medium purged with 95% O_2_ and 5% CO_2_ in the A/R and A/R intervention groups. Cells in the control group were perfused with medium saturated with 95% air and 5% CO_2_. Immediately after wash-out of the DCFH_2_-DA incubation medium (~10 s), cells were visualized with a stereo fluorescence microscope (λ_ex_ = 470 ± 20 nm, λ_em_ = 515 nm long pass; model M165 C, Leica Microsystems, Wetzlar, Germany) for a period of 30 min at 2.5-min intervals with dark intermittence periods. Cellular DCF fluorescence was subsequently quantified based on total pixel intensity of the images following conversion to 8-bit grayscale (ImageJ software; National Institutes of Health, Bethesda, MD, USA) and normalized to t = 0 min.

### 2.12. Statistical Analysis

Statistical analysis was performed using GraphPad Prism (GraphPad Software, La Jolla, CA, USA). All data were analyzed using one-way ANOVA, in which repeated-measures data were analyzed based on area under the curve (AUC) values per sample. Intragroup multiple comparisons were made using the Dunnet’s post-hoc test, where means were compared to a single control. For intergroup multiple comparisons, all possible pairs of means were compared using Tukey’s range test. A *p*-value of < 0.05 was considered statistically significant.

## 3. Results

### 3.1. The Spectral Properties of DCFH_2_-DA and Derivatives Are pH-Dependent

The spectral properties of DCFH_2_-DA, DCFH_2_, and DCF were analyzed using absorbance and fluorescence spectroscopy, the results and discussion of which are provided in Section S3.1 and Figures S4–S6. The most important and relevant finding was that changes in pH (up to pH = 8.0) affect the absorption spectrum and fluorescence emission and excitation spectra of DCF. Therefore, changes in pH are likely to affect experimental data interpretation and outcomes, particularly under conditions of acidosis.

### 3.2. The Stability of DCFH_2_-DA and DCFH_2_ in Aqueous Solvent and Medium Is Dependent on the Composition of the Solution

**Figure 2:**
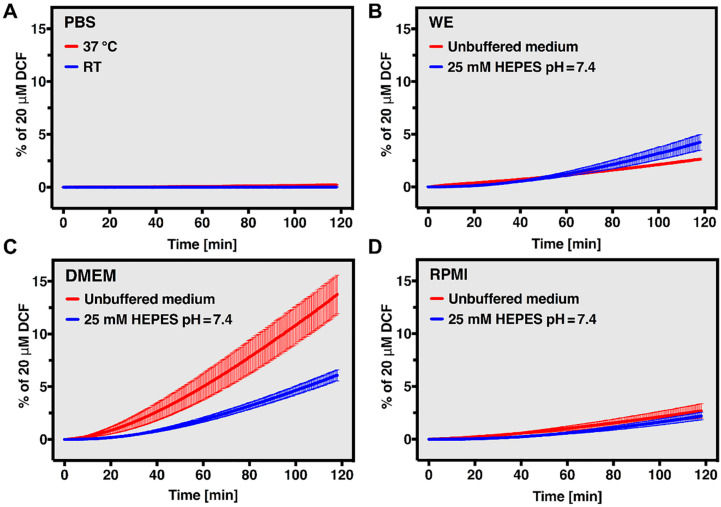
Stability of DCFH_2_-DA in PBS and medium. (**A**) Stability of 20 μM DCFH_2_-DA in PBS measured at room temperature (RT; blue line) or 37 °C (red line) over a period of 2 h. DCFH_2_-(DA) stability was measured by means of DCF formation and plotted as a percentage of a 20-μM DCF reference sample. (**B**–**D**) Stability of 20 μM DCFH_2_-DA in (**B**) unbuffered (red line) and HEPES-buffered (25 mM, pH = 7.4; blue line) William’s E (WE), (**C**) Dulbecco’s modified Eagle medium (DMEM), and (**D**) Roswell Park Memorial Institute (RPMI) medium measured at 37 °C over a period of 2 h. DCFH_2_-(DA) stability was measured by means of DCF formation and plotted as a percentage of a 20-μM DCF reference sample. All data (n = 3/group) are plotted as mean ± SD.

The long- and short-term stability of DCFH_2_-DA and DCFH_2_ was analyzed in aqueous solvents (discussed in the Supplemental Information) and cell culture media (the latter only for DCFH_2_-DA). Decay of DCFH_2_-DA in aqueous solution, resulting in the formation of DCF, most likely occurs through base-catalyzed hydrolysis of the acetate groups and subsequent DCFH_2_ (auto-)oxidation. DCF generation as a measure for DCFH_2_-DA decay was analyzed spectrofluorometrically in solutions containing 20 μM DCFH_2_-DA and plotted as percentage of a 20-μM DCF reference sample (Figure 2).

Temperature- and solute-related effects were observed when the short-term stability of DCFH_2_-DA was determined in different types of cell culture media (WE, DMEM, and RPMI-1640) and PBS; conditions that are relevant for in vitro use of the probe. DCF formation in PBS was undetectable at RT and negligible at 37 °C following 2 h of incubation (Figure 2A), which is in line with the data on long-term stability of DCFH_2_-DA in aqueous solvent (Figure S7). DCFH_2_-DA stability in cell culture medium (37 °C) was analyzed at ambient CO_2_ tension in serum- and phenol red-free DMEM, RPMI, and WE with or without HEPES (25 mM, pH = 7.4). The reason for this was that microplate-based experiments involving DCFH_2_-DA (as described below) generally need to be performed under these conditions. Considerable variation in the extent of DCF formation was noted for the different types of culture medium and depending on the presence of HEPES. Given that HEPES prevents medium alkalization at ambient CO_2_ tension, its presence was expected to improve DCFH_2_-DA stability due to a reduction in [OH^−^]-mediated deacetylation. However, addition of HEPES to the different medium formulations resulted in opposing effects on DCF formation. Specifically, the presence of HEPES deterred DCF formation in WE (Figure 2B), favored it in DMEM (Figure 2C), and had no noticeable effect in RPMI (Figure 2D). Considering that the influence of HEPES on DCF formation appears to be independent of its presumed inhibitory effect on DCFH_2_-DA deacetylation, the observed differences most likely result from complex interactions between DCFH_2_, HEPES, and redox-active medium components (Table 1). In that regard, the extent of DCF formation in DMEM corresponds well to it being the most nutrient-rich medium with a high concentration of O_2_^•−^-producing riboflavin, granted that (room) light is present to facilitate this reaction [34]. DMEM is moreover the only medium containing Fe^3+^ that, together with O_2_^•−^, will result in the formation of redox-active Fe^2+^ through Haber-Weiss cycling [35].

Although all types of media contain varying amounts of antioxidants (e.g., GSH and ascorbic acid), these radical-scavenging compounds become radicals themselves upon oxidation [7]. Hence, ‘antioxidants’ can also function as pro-oxidants under the appropriate circumstances. Accordingly, the free radical scavengers GSH [36], cysteine [36], and ascorbic acid [37] were all shown to generate H_2_O_2_ in DMEM and/or RPMI. Addition of another compound with similar properties, in this case HEPES [38,39], could therefore shift the redox equilibrium of the sample in opposing ways. As a result, the extent to which DCFH_2_ oxidation takes place will strongly depend on medium composition. The addition of HEPES to WE, for instance, could result in HEPES reacting with ascorbic acid radicals (formed as a result of, e.g., O_2_^•−^ generation by riboflavin [40,41]) that would otherwise be converted into redox-unreactive dehydroascorbic acid. Consequently, HEPES would function as a catalyst for O_2_^•−^ formation [38] and thereby enhance DCFH_2_ oxidation (Figure 2B). In DMEM, which contains a large portion of oxidant-generating compounds together with low levels of antioxidants, HEPES will likely act as a free-radical sink, consequently reducing the formation of DCF (Figure 2C).

In conclusion, although DCFH_2_-DA is relatively stable in aqueous solution when kept at 4 °C for up to 24 h, long-term storage in water or physiological buffers is not recommended. It is also advisable to take the effects of cell culture media components into consideration when performing in vitro assays using DCFH_2_-DA, and preferably not use DMEM.

### 3.3. DCFH_2_-DA Rapidly Accumulates in HepG2 and HepaRG Cells

**Figure 3:**
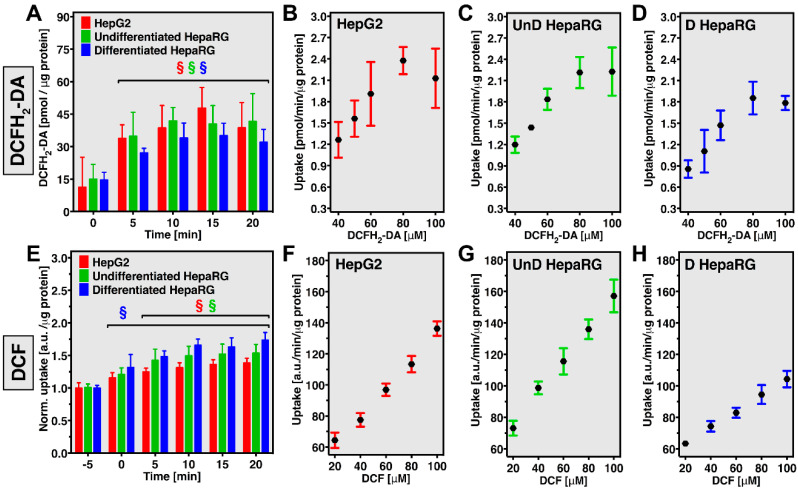
Time-dependent uptake and cellular uptake rates of DCFH_2_-DA and DCFH_2_ by HepG2 and HepaRG cells. (**A**) Time-dependent uptake of 50 μM DCFH_2_-DA (pmol/μg protein) by HepG2 (red bars), undifferentiated (green bars), and differentiated HepaRG cells (blue bars). DCFH_2_-DA uptake rates (pmol/min/μg protein) for (**B**) HepG2, (**C**) undifferentiated, and (**D**) differentiated HepaRG cells, determined for 40–100 μM DCFH_2_-DA. (**E**) Time-dependent uptake of 50 μM DCF (a.u./μg protein) by HepG2 (red bars), undifferentiated (green bars), and differentiated HepaRG cells (blue bars). DCF uptake rates (a.u./min/μg protein) for (**F**) HepG2, (**G**) undifferentiated, and (**H**) differentiated HepaRG cells, determined for 20–100 μM DCF. All data (n = 8/group) are plotted as mean ± SD. Statistical significance (*p* < 0.05) is designated ‘§’ for intragroup analyses.

Uptake of DCFH_2_-DA as a function of time and concentration was analyzed in HepG2 and undifferentiated as well as differentiated HepaRG cells (Figure 3). Time-dependent uptake of DCFH_2_-DA was rapid, as evinced by the presence of detectable amounts of DCFH_2_-DA at t = 0 min (i.e., very brief contact between the monolayer and the incubation medium), and plateaued at t = 5 min in all groups (Figure 3A). These data indicate that a DCFH_2_-DA incubation time of 5–10 min suffices for single-read experiments. The findings are consistent with reports on the uptake of DCFH_2_-DA by Chinese hamster ovary cells [16] and on the hepatocellular uptake of 5(6)-carboxy-2′7′-dichlorofluorescein diacetate (carboxy-DCF-DA) [42]. Passive diffusion is generally assumed to be the main uptake mechanism for DCFH_2_-DA [43], as was demonstrated by linear uptake of carboxy-DCF-DA (measured intracellularly through its deacetylation product carboxy-DCF) over a 0–500-μM range [42]. In contrast, the DCFH_2_-DA uptake rate in HepG2 and HepaRG cells deviated from linearity at 100 μM (Figure 3B–D), which might indicate a greater role for uptake through plasma membrane transporters such as organic anion transporting proteins 1B1 and 1B3 (OATP1B1 and OATP1B3, respectively) [44]. However, saturation of hepatocellular carboxy-DCF uptake, which strongly depended on OATP activity, did not occur until concentrations exceeded 100 μM [42]. Finally, the intracellular accumulation of carboxy-DCF was significantly decreased at 4 °C compared to 37 °C incubation (at 4 °C the rate of endocytosis and enzymatic/transporter activity is vastly reduced), whereas uptake of its acetylated derivate was temperature-independent [42]. Accordingly, transporter-mediated DCFH_2_-DA uptake is likely not a major contributor to probe accumulation.

Saturation of cytosolic esterase activity could form an alternative explanation for the observed plateau in the DCFH_2_-DA uptake rate. Assuming that cellular DCFH_2_-DA uptake mainly proceeds through passive diffusion, intracellular [DCFH_2_-DA] will not exceed extracellular [DCFH_2_-DA], i.e., that in the incubation medium. The concomitant esterase-dependent intracellular formation of DCFH_2_ however establishes a DCFH_2_-DA sink that allows for ongoing probe influx. Thus, when this enzymatic process becomes saturated, the concentration gap between intracellular and extracellular [DCFH_2_-DA] will stabilize as a result of which probe uptake will concomitantly not increase at higher probe concentrations. This principle has been shown for acetylsalicylate uptake by hepatocytes co-incubated with the acetylesterase inhibitor paraoxon [45]. The presumed acetylesterase-dependency of DCFH_2_-DA uptake could further explain the differences in uptake rate between, e.g., HepG2 and differentiated HepaRG cells (Figure 3B,D), and underscores the notion that DCFH_2_-DA accumulation is likely cell-type specific.

### 3.4. DCF Accumulates in HepG2 and HepaRG Cells and Is Poorly Retained

The cellular uptake and excretion of DCF were analyzed because such effects are expected to skew experimental data in an upward or downward direction, respectively, when using DCFH_2_-DA to assay intracellular oxidative stress. DCF uptake was determined in a time- and concentration-dependent manner. The fluorophore accumulated swiftly in all cell types, yet more rapidly in differentiated HepaRG cells compared to HepG2 and undifferentiated HepaRG cells (Figure 3E), as evidenced by significant accumulation at t = 0 min in differentiated HepaRG cells compared to t = 5 min in HepG2 and undifferentiated HepaRG cells. Accordingly, overall DCF uptake was higher in differentiated HepaRG cells (*p* < 0.0001 and 0.001 compared to HepG2 and undifferentiated HepaRG cells, respectively, at t = 20 min) as well as in undifferentiated HepaRG compared to HepG2 cells (*p* < 0.01 at t = 20 min). This trend roughly corresponds to RNA expression levels of OATP1B1 and OATP1B3 that followed differentiated HepaRG > undifferentiated HepaRG >> HepG2 cells [44]. Insofar as culture conditions for differentiated HepaRG cells differed between the referenced study and this study, these results might not be fully superimposable. Considering that OATP1B1 and OATP1B3 are involved in the hepatocellular uptake of carboxy-DCF [42] as well as fluorescein [25,46], both are likely to transport DCF as well. Although DCF is generally assumed to be membrane-impermeable, uptake through passive diffusion could also take place (as discussed below). These findings are of particular relevance for experiments in which prolonged incubation with DCFH_2_-DA is desired because considerable DCF formation from DCFH_2_-DA decay occurs under such conditions (Section 3.2, Figure 2).

The DCF uptake rate was linear over the complete concentration range in all cell types (Figure 3F–H), in accordance with data on carboxy-DCF showing that saturation occurs at [DCF] > 100 μM [42]. In contrast to time-dependent DCF uptake (Figure 3E), the DCF uptake rate was lower in differentiated HepaRG cells compared to HepG2 and undifferentiated HepaRG cells. Time-dependent DCF uptake was measured in cell lysates, whilst the DCF uptake rate was analyzed in intact monolayer culture. This difference in experimental setup opens up the possibility that the overall probe distribution over the complete sample (i.e., cells and incubation medium) may differ, which might affect DCF fluorescence emission properties. Nevertheless, the data collectively show that DCF formation in the incubation medium could interfere with intracellular measurements and that the extent of this effect is cell-type specific.

**Figure 4:**
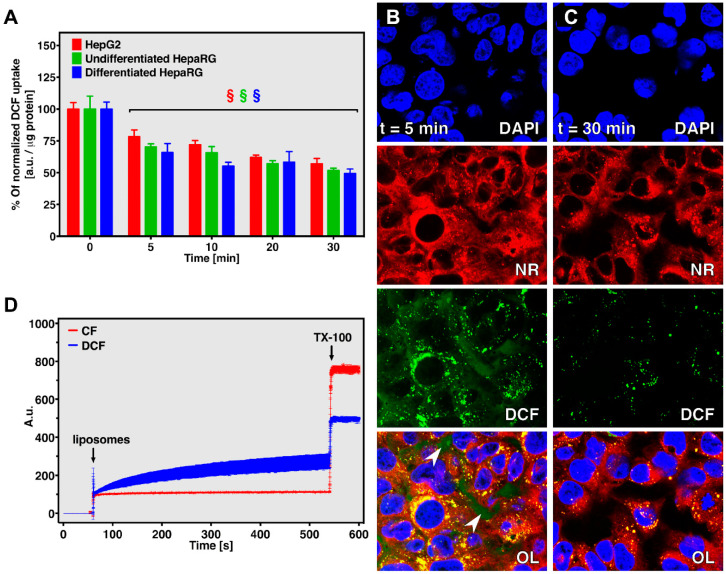
DCF retention in cells and liposomes. (**A**) DCF retention (a.u./μg protein) was measured in HepG2 (red bars), undifferentiated HepaRG cells (green bars), and differentiated HepaRG cells (blue bars) loaded with 100 μM DCF for 2 h. Data (n = 8/group) are plotted as mean ± SD. Intragroup statistical differences (*p* < 0.05) are designated with ‘§’. (**B**,**C**) Intracellular and extracellular localization of DCF in HepG2 cells loaded with 100 μM DCF for 2 h following (**B**) t = 5 min and (**C**) t = 30 min of incubation in probe-free medium. Cells were counterstained with DAPI (nucleus) and Nile Red (NR; membranes). OL: overlay. The arrowheads in (**B**, overlay) point to the extracellular presence of DCF. (**D**) Efflux of DCF (blue line) and CF (red line) from liposomes encapsulating the probes at self-quenching concentration was analyzed spectrofluorometrically (λ_ex_ = 491 ± 5 nm and λ_em_ = 523 ± 5 nm) in time-based acquisition mode (t = 600 sec) at 37 °C. At t = 550 s, 0.15% Triton X-100 (TX-100) was added to disrupt the liposomal membrane and release all encapsulated probe. Data (n = 3) are plotted as mean ± SD.

Intracellular DCF retention was quantified spectrofluormetrically and visualized using confocal microscopy. A rapid reduction in DCF fluorescence, i.e., probe efflux, was observed for all cell types. DCF efflux stabilized at t = 20 min, at which point ~50% of initial probe fluorescence was detected (Figure 4A). DCF expulsion occurred more rapidly in differentiated HepaRG cells compared to HepG2 cells (*p* < 0.001 at t = 5 min) and undifferentiated HepaRG cells (*p* < 0.001 at t = 10 min). As for DCF uptake and OATP1B1/3, this trend fits well with RNA expression levels of the basolateral exporter multidrug resistance protein 3 (MRP3) that followed differentiated HepaRG > undifferentiated HepaRG >> HepG2 cells [44], although differences in culture medium composition for the differentiated HepaRG cells need to be taken into consideration. Although both MRP3 and the apically located MRP2 expel carboxy-DCF from hepatocytes in vivo [42], MRP2 likely does not contribute to DCF efflux in monolayer culture because of its cytosolic localization under these conditions [47]. Correspondingly, MRP2 mRNA expression levels were comparable between all cell types [44].

Efflux of DCF into the extracellular space was confirmed using confocal microscopy of HepG2 cells that were loaded with 100 μM DCF prior to imaging at t = 5 and 30 min following replacement of the incubation solution with probe-free WE medium (Figure 4B,C, respectively). At t = 5 min, DCF was already localized both intracellularly and in the extracellular spaces (indicated by white arrows in Figure 4B, overlay panel). The co-localization between DCF and Nile Red indicates that DCF accumulates in organelles. Considering that the hepatic metabolism of fluorescein proceeds through glucuronidation [48], it is plausible that DCF is processed similarly and therefore localizes in the endoplasmic reticulum. Significant loss of intracellular and extracellular DCF fluorescence was observed at t = 30 compared to t = 5 min, supporting the notion that DCF is actively excreted by HepG2 cells. Glucuronidation of DCF might additionally contribute to the observed loss in fluorescence since the fluorescence intensity of glucuronidated fluorescein constitutes only 4.5% of that of unconjugated fluorescein [48], although further work is needed to substantiate this.

In conclusion, DCF uptake and efflux both occur in HepG2 and HepaRG cells, presumably through transporter-mediated mechanisms. Movement of DCF across the plasma membrane needs to be taken into account when performing assays involving DCFH_2_-DA on these cell types, as it is expected to affect experimental outcomes.

### 3.5. DCF Crosses Membranes

The membrane-crossing ability of DCF was investigated by means of liposomes encapsulating DCF and the more hydrophilic 6-carboxyfluorescein (CF) at self-quenching concentrations, a system that generates fluorescence upon probe leakage [31]. An increase in fluorescence (i.e., probe efflux) was observed directly after the addition of DCF-encapsulating liposomes to the iso-osmotic buffer solution, something that was not observed for CF-encapsulating liposomes (Figure 4C). CF differs from DCF in that it lacks the two chlorine moieties but has an additional carboxylic acid group, giving it an overall charge of −3 at physiological pH [49] compared to −2 for DCF (Figure S8 and Figure 1) [14]. It is likely that this difference in charge explains the passage of DCF, but not CF, across the negatively charged phospholipid bilayer that normally deters the diffusion of negatively charged small molecules [50]. Despite providing information on the ability of DCF to cross phospholipid membrane, the experimental setup employed here greatly differs from in vitro conditions. Most relevantly, the high intraliposomal DCF concentration (18 mM) creates a nonphysiological but significant concentration gradient that potentially favors DCF efflux even at iso-osmotic conditions.

### 3.6. Basal Oxidant Formation and Cellular Metabolic Rate Differ between HepG2 and HepaRG Cells

**Figure 5:**
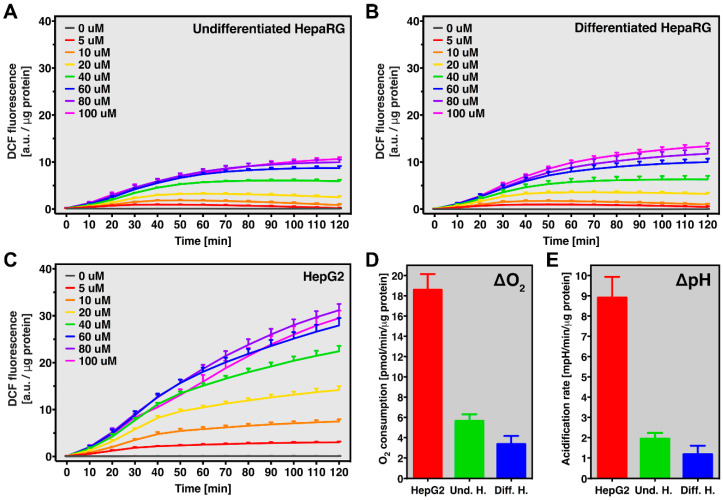
Basal oxidant formation and cellular metabolic rate in HepG2 and HepaRG cells. DCF fluorescence (a.u./μg protein) over time at increasing DCFH_2_-DA concentration (0–100 μM) as a measure of basal oxidant formation in (**A**) resting undifferentiated HepaRG, (**B**) differentiated HepaRG cells, and (**C**) HepG2 cells. Data (n = 6/group) are presented as mean ± SD. (**D**) Cellular O_2_ consumption (pmol/min/μg protein) and (**E**) extracellular acidification rates (mpH/min/μg protein) of HepG2 (red bars), undifferentiated HepaRG cells (Und. H.; green bars), and differentiated HepaRG cells (Diff. H.; blue bars). Data (n = 8/group) are presented as mean ± SD.

The rate of basal oxidant production over time was analyzed at increasing concentrations of DCFH_2_-DA in HepG2 and undifferentiated as well as differentiated HepaRG cells to ascertain the optimal probe concentration for assays involving these cell types. DCF formation (as a measure for intracellular oxidant formation) correlated to DCFH_2_-DA concentration up to 60 μM in all cell types (Figure 5A–C), indicating that intracellular probe levels likely reach saturating conditions at ≥ 60 μM in resting cells. Overall, DCF formation was significantly higher in HepG2 cells compared to undifferentiated and differentiated HepaRG cells (*p* < 0.001 following 2 h incubation at 80 μM for both groups), likely due to a more pro-oxidant redox state in HepG2 cells. This notion is supported by the data on cellular O_2_ consumption and extracellular acidification rate, which suggest a higher metabolic rate, more glycolytic state, and/or differences in uncoupling processes in HepG2 cells compared to both types of HepaRG cells (Figure 5D,E; *p* < 0.001 for both groups). Accordingly, glucose consumption and lactate production are higher in HepG2 cells compared to (differentiated) HepaRG cells [51]. Nevertheless, the difference in DCF formation between HepG2 and HepaRG cells could also, in part, derive from variations in DCF metabolism that can affect DCF fluorescence intensity as described above. Antioxidant chemotypes could further differ between the cell lines and in this way lead to a difference in DCF formation. Gene expression for common antioxidant proteins did not greatly differ between the cell lines, with the exception of peroxiredoxin-6 and catalase, which were in the order of differentiated and undifferentiated HepaRG > HepG2 cells [44]. Overall, the extent of basal oxidant formation in resting HepG2 and HepaRG cells roughly correlates to lactate formation and O_2_ consumption and is best measured at a DCFH_2_-DA concentration of ≥ 60 μM.

### 3.7. Oxidative Stress during In Vitro Anoxia/Reoxygenation Can Be Visualized in Real-Time Using DCFH_2_-DA

**Figure 6:**
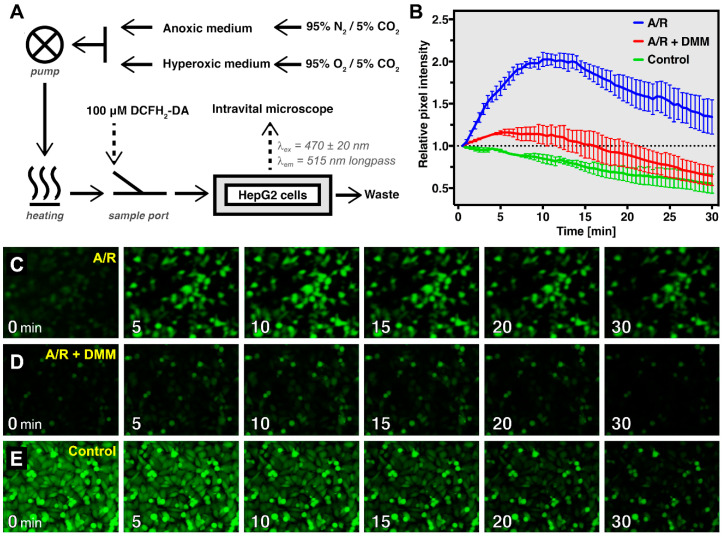
Real-time visualization of oxidative stress during in vitro anoxia-reoxygenation (A/R). (**A**) Schematic overview of the perfusion setup under conditions of A/R; details are provided in the text. (**B**) Formation of DCF (relative pixel intensity) from DCFH_2_-DA (100 μM) in HepG2 cells during 30 min of reoxygenation following 4 h of anoxia and incubation with DCFH_2_-DA under anoxic conditions (A/R, blue line) or similar procedures in the presence of the antioxidant dimethyl malonate during the anoxia period (A/R + DMM, red line). Control cells were perfused and incubated with DCFH_2_-DA under normoxic conditions (4 h perfusion followed by 30 min imaging; control, green line). Data (n = 3/group) are presented as mean ± SEM. Representative fluorescence microscopy images of the data presented in (**B**) are shown for reperfused cells following (**C**) anoxia, (**D**) reperfusion following anoxia + DMM, and (**E**) control conditions.

DCFH_2_-DA was used to visualize and quantify acute oxidative stress in HepG2 cells subjected to 4 h of anoxia followed by reoxygenation (A/R) in a perfusion setup equilibrated to 37 °C and positioned under a fluorescence microscope (Figure 6A and Figure S3). Control cells were exposed to standard culture conditions, i.e., medium saturated with 95% air and 5% CO_2_, throughout the experiment. A rapid and significant increase in DCF fluorescence was observed in the cells subjected to A/R (Figure 6B,C). DCF fluorescence intensity reached a maximum at 10–12 min of reoxygenation and subsequently declined, presumably due to a reduced rate of DCF formation at constant DCF efflux and possibly due to metabolism. Considering that hepatocytes exposed to anoxia become acidic [52], the observed increase in DCF formation in this early phase of reoxygenation could well be underestimated due to reduced DCF fluorescence emission at pH < 7 (Figure S6D). In comparison, a decrease in DCF fluorescence was observed in control cells following incubation with DCFH_2_-DA despite higher fluorescence at baseline (Figure 6C,E). This phenomenon is attributable to the difference in incubation conditions between the control and A/R group, i.e., oxygenated vs. deoxygenated medium, which enabled DCFH_2_ oxidation to take place in control cells but not in A/R cells during the incubation period.

Addition of 1 mM DMM to the perfusion and incubation medium resulted in a profound decrease in DCF formation during reoxygenation (Figure 6C,D). Considering that the effect of DMM stems from a reduction in succinate accumulation during anoxia that hampers mitochondrial oxidant formation upon reoxygenation [33], the increase in DCF fluorescence during A/R most likely resulted from mitochondrial oxidative stress that manifests in the cytosol where mitochondria-derived oxidants react with DCFH_2_. In that respect, the maximum signal intensity observed at t ~10 min indicates that mitochondrial oxidant formation during A/R is a rapid yet short-lived effect, as has previously been postulated in the context of hepatic ischemia-reperfusion [32].

## 4. Discussion

**Figure 7:**
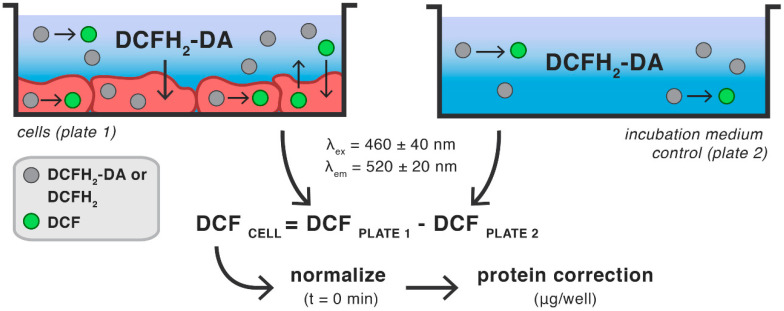
Schematic overview of optimized assay conditions for the use of DCFH_2_-DA in cultured hepatocytes. Details are provided in the text.

The main limitations for the use of DCFH_2_-DA in experiments with HepG2 and HepaRG cells are uptake of extracellular DCF, poor DCF retention, and cell type-specificity of DCFH_2_ oxidation under resting conditions. DCFH_2_-DA stability is notably affected by the type and pH of incubation medium in which it is dissolved. Nevertheless, DCFH_2_-DA can be a useful means to analyze general oxidative stress in HepG2 and HepaRG cells granted that the experimental setup accounts for these limitations. A schematic overview of an experimental setup that aims to optimize conditions for the use of DCFH_2_-DA in cells of hepatocellular origin is presented in Figure 7.

The incubation medium should preferably contain ≥ 60 μM DCFH_2_-DA in either PBS or serum-free WE or RPMI medium, which needs to be HEPES-buffered when performing experiments at ambient CO_2_ tension to maintain pH. The assay is ideally performed in a fluorescence microplate reader (equilibrated to 37 °C) in time-based acquisition mode and equipped with the appropriate filters for DCF fluorescence detection (e.g., λ_ex_ = 460 ± 40, λ_em_ = 520 ± 20 nm). Directly following analysis of the assay plate, a second plate containing only incubation medium (exactly corresponding to that used in the assay plate) should be read to correct for extracellular DCF formation. The raw data from the cell-free plate (2) need to be subtracted from the corresponding wells of the assay plate (1) at each individual time point to calculate cellular DCF fluorescence (Figure 7, equation). Sample a.u. values should be close to 0 for the baseline measurement following this step, as was the case with the data presented in Figure 5. However, if considerable differences in baseline values prevail, for instance due to a significant time lag in pipetting the incubation medium, data can be additionally normalized by subtracting the raw data at baseline (first read) from every subsequent time point per sample (Figure 7, normalize). Irrespective of whether baseline correction is performed, each data point should be normalized to the total protein (or DNA) content per well to correct for differences in seeding density (Figure 7, protein correction).

By incubating the cells with DCFH_2_-DA throughout the assay period, intracellular DCFH_2_(-DA) levels are maintained constantly. Moreover, intracellularly generated DCF that is expelled into the extracellular space will be detected, whilst outcomes are corrected for DCF formation in the incubation medium. In doing so, DCFH_2_-DA can be used to generate data on intracellular oxidative stress with minimal interference. This approach could be useful when DCFH_2_-DA, as well as other fluorogenic probes with similar properties (e.g., dihydrorhodamine 123 and hydroethidine [53]), are used on cell lines that express membrane transport proteins, such as MRP1-expressing RAW 264.7 macrophages [53].

There are limitations to DCFH_2_-DA that are more cumbersome to correct for. Specifically, DCFH_2_ oxidation involves the formation of an intermittent radial species, the DCF semiquinone radical (DCF^•^/DCF^•−^) [13,14], which could affect DCF formation via both direct, i.e., DCFH_2_ oxidation, and indirect, i.e., O_2_^•−^ formation, mechanisms [7,13]. In addition, overoxidation of DCF into nonfluorescent degradation products could lead to an underestimation of DCF formation [7]. Although these phenomena are not specific to DCFH_2_-DA [7], it will be difficult to quantify the full extent to which these processes contribute to DCF formation under assay conditions and to correct for them experimentally. A second, more relative limitation of DCFH_2_(-DA) is its non-specificity towards various radical and non-radical oxidants [9,10,11,12]. It is therefore suggested to regard data generated using DCFH_2_-DA as a measure for overall ‘oxidative’ or ‘redox stress’ and to avert to more specific probes (e.g., hydroethidine or Amplex Red) or techniques (e.g., electron paramagnetic resonance) when detection of particular radical or oxidant species is warranted.

In addition to the experimental setup presented in Figure 7, the probe can be also used to visualize and quantify cytosolic oxidative stress during A/R in real time. This technique can for example be employed to analyze the direct effect of pharmacological interventions that target intracellular oxidative stress or to analyze other phenomena using different dyes (e.g., JC-1 for mitochondrial membrane potential analysis), further expanding the possible applications of DCFH_2_-DA.

## 5. Conclusions

Despite its limitations DCFH_2_-DA can function as a useful fluorogenic probe to investigate the intracellular redox state in the context of liver disease. Experimental setups are proposed that circumvent most of the interfering factors. Nevertheless, caution should always be taken when interpreting data generated using fluorogenic redox probes as it is not possible to correct for all limitations.

## Figures and Tables

**Table 1:** Redox-active compounds in common cell culture media. All compounds are listed in μM concentration, calculated from the manufacturer’s data sheets (Table S1).

		DMEM	RPMI	WE
**Salts**	Fe^3+^ (nitrate)	0.25	0.00	0.00
**Vitamins**	Ascorbic acid	0.00	0.00	11.36
	Riboflavin	1.06	0.53	0.27
**Amino acids**	Cysteine	0.00	0.00	330.17
	Histidine	270.69	96.67	96.67
	Methionine	201.06	100.53	100.53
	Phenylalanine	399.54	90.80	151.34
	Tryptophan	78.34	24.48	48.96
	Tyrosine	397.37	110.38	193.17
**Other compounds**	GSH	0.00	3.25	0.16

## Data Availability

Data is contained within the article and supplementary material.

## Supplementary Materials

The following are available online at https://www.mdpi.com/article/10.3390/antiox10050674/s1, Table S1, List of chemicals and reagents; Figure S1, optimization of BCA protein determination—cell lysis buffers; Figure S2, optimization of BCA protein determination—Triton X-100 concentration; Figure S3, perfusion setup for anoxia/reoxygenation experiments; Figure S4, absorption spectra and molar extinction coefficients of DCF and DCFH2; Figure S5, absorption spectra and molar extinction coefficients of DCFH2-DA; Figure S6, pH-dependent spectral properties DCFH2 and DCF; Figure S7, stability of DCFH2 and DCFH2-DA in aqueous solution; Figure S8, chemical structure of CF and DCF.
