# Supplementary material for: Analysis and Optimization of Conditions for the Use of 2′,7′-Dichlorofluorescein Diacetate in Cultured Hepatocytes
Source: Antioxidants (Basel). 2021 Apr 26;10(5):674. doi: 10.3390/antiox10050674 (PMC8147027; doi:10.3390/antiox10050674)
Supplement: Supplementary file 1 [file antioxidants-10-00674-s001.zip › antioxidants-1132658-supplementary.pdf]

SUPPLEMENTAL INFORMATION

Analysis and optimization of conditions for the use of 2´,7´-dichlorofluorescein diacetate on hepatocyte cell lines

Megan J. Reiniers[1,2], Lianne R. de Haan[1,3], Laurens F. Reeskamp[4], Mans Broekgaarden[5,6], Ruurdtje Hoekstra[7], Rowan F. van Golen[8], Michal Heger[1,3,9]

[1] Department of Pharmaceutics, College of Medicine, Jiaxing University, Jiaxing, Zhejiang, P. R. China

[2] Department of Surgery, Haaglanden Medisch Centrum, the Hague, the Netherlands

[3] Laboratory Experimental Oncology, Department of Pathology, Erasmus MC, Rotterdam, the Netherlands

[4] Department of Vascular Medicine, Amsterdam UMC, Location AMC, Amsterdam, the Netherlands

[5] Team Cancer Targets and Experimental Therapeutics, Department Microenvironment Cell Plasticity and Signaling, Institute for Advanced Biosciences, Université de Grenoble-Alpes, Allée des Alpes, La Tronche, France

[6] INSERM U 1209, CNRS UMR 5309, Allée des Alpes, La Tronche, France

[7] Tytgat Institute for Liver and Intestinal Research, Amsterdam UMC, Location AMC, Amsterdam, the Netherlands

[8] Department of Gastroenterology and Hepatology, Leiden University Medical Center, Leiden, the Netherlands

[9] Department of Pharmaceutics, Utrecht Institute for Pharmaceutical Sciences, Utrecht University, Utrecht, the Netherlands

S2. Methods

S2.1. Reagents and buffers

Table S1. List of chemicals and reagents used in the main manuscript.

**Compound**	**Purity**	**Supplier**	**Additional information**
1,2-dimyristoyl-*sn*-glycero-3-phosphocholine (DMPC)	> 99%	Avanti Polar Lipids$	Dissolved in $CHCl_3$
1,2-dioleoyl-*sn*-glycero-3-phosphoethanolamine-N-lactosyl (18:1 lactosyl PE)	> 99%	Avanti Polar Lipids$	Dissolved in $CHCl_3$, stored under N_2 at -20 °C
Accutase	n/a	Sigma-Aldrich#	
Accumax	n/a	Sigma-Aldrich#	
5,6-Carboxyfluorescein	≥ 95%	Sigma-Aldrich#	
$CHCl_3$	≥ 99%	Sigma-Aldrich#	
Cholesterol	> 98%	Avanti Polar Lipids$	Dissolved in $CHCl_3$, stored under N_2 at -20 °C
Collagen I solution	n/a	Sigma-Aldrich#	From rat tail
Dimethyl malonate	98%	Sigma-Aldrich#	
DMSO	≥ 99.5%	Merck Millipore*	
Dulbecco's modified Eagle medium	n/a	Lonza†	Phenol red-free
Fetal bovine serum	n/a	Lonza†	
Ganglioside GM1	> 99%	Avanti Polar Lipids$	Dissolved in $CHCl_3$:CH_3OH:H_2O [65:35:8, v:v], stored under N_2
HEPES (Na-salt)	≥ 99.5%	Sigma-Aldrich#	
Human recombinant insulin solution	≥ 27 U/mg	Sigma-Aldrich#	
Hydrocortisone 21-hemisuccinate	n/a	Sigma-Aldrich#	
L-glutamine	n/a	Lonza†	
MeOH	≥ 99.9%	Sigma-Aldrich#	
Triton X-100	≥ 99.95%	Sigma-Aldrich#	
NaOH	≥ 99%	Sigma-Aldrich#	
NaCl	≥ 99%	Sigma-Aldrich#	
Phosphate-buffered saline	n/a	Fresenius Kabi§	
Penicillin/streptomycin	n/a	Lonza†	
Roswell Park Memorial Institute medium 1640 (RPMI-1640)	n/a	Lonza†	Phenol red-free
Water	Milli-Q	Merck Millipore&	18.2 MΩ · cm at 25 °C
William's E medium	n/a	Lonza†	Phenol red-free

* Darmstadt, Germany
# St. Louis, MO
† Basel, Switzerland
$ Alabaster, AL
§ Bad Homburg, Germany
& Billerica, MA

S2.7. Cellular $DCFH_2$-DA uptake

The bicinchoninic acid (BCA) assay is commonly employed to determine protein content. The reaction relies on the reduction of Cu^{2+} to Cu^{+} by peptide bonds in the protein, which occurs at stoichiometric proportions. The Cu^{+} subsequently chelates two BCA molecules to form a chromophore that can be measured spectrophotometrically [1].

To determine the impact of various cell lysis solvents on the extraction efficiency and the BCA chromogenic reaction, the BCA assay was performed on albumin as model protein diluted in the following cell lysis solutions (all in water): (1) 1 mM boric acid; (2) 1 mM boric acid + 20% (v/v) methanol (MeOH); (3) 0.2 M sodium hydroxide (NaOH); (4) 0.2 M NaOH + 20% MeOH; (5) 1 M formic acid; (6) 1 M formic acid + 20% MeOH; (7) 0.1% (w/v) sodium dodecyl sulfate (SDS); (8) 0.1% SDS + 20% MeOH; (9) 0.1% Triton X-100; (10) 0.1% Triton X-100 + 20% MeOH; (11) water; (12) water + 20% MeOH.

Albumin (fraction V, ≥ 96%, Sigma-Aldrich, St. Louis, MO) was dissolved in water at a 2 mg/mL stock concentration. A serial titration series was prepared in water, such that the final protein concentrations after 4-fold dilution in the cell lysis solution were 500-, 250-, 125-, 62.5-, 31.75-, and 0 µg/mL. The titration in 0.2 M NaOH was made in incremental steps of 100 µg/mL as exception because these standards had already been prepared and were used up during the experiment. The BCA assay was performed according to the manufacturer's instructions in a 96-well plate (Greiner Bio-One, Kremsmünster, Austria). Absorbance was measured at 540 nm in a plate reader (Synergy HT, Biotek Instruments, Winooski, VT).

To determine the effect of Triton X-100 concentration on the BCA reaction, the experiments were repeated as described above with cell lysis solution containing the following Triton X-100 concentrations: 5%, 2.5%, 1%, 0.5%, 0.1%, and 0%. Subsequently, protein extraction was performed on cultured HepG2 cells to determine which concentration Triton X-100 produced the greatest protein

yield (n = 3 per Triton X-100 concentration). HepG2 cells were cultured in 24-well plates (Greiner Bio-One) as described in 'Cell culture' in the main text. Next, 300 µL of cell lysis solution was added per well, the plate was placed on a rocker for 30 min, and protein content was determined according to the standard operating procedure provided by the manufacturer. A standard curve was prepared using albumin. Protein concentrations were extrapolated from the linear fit function of the standard curve.

All data were processed in Microcal Origin (OriginLab, Northampton, MA) and fitted with a linear fit to determine the fit function and the goodness of fit (R^2 value). The results are presented in Figure S1 and S2.

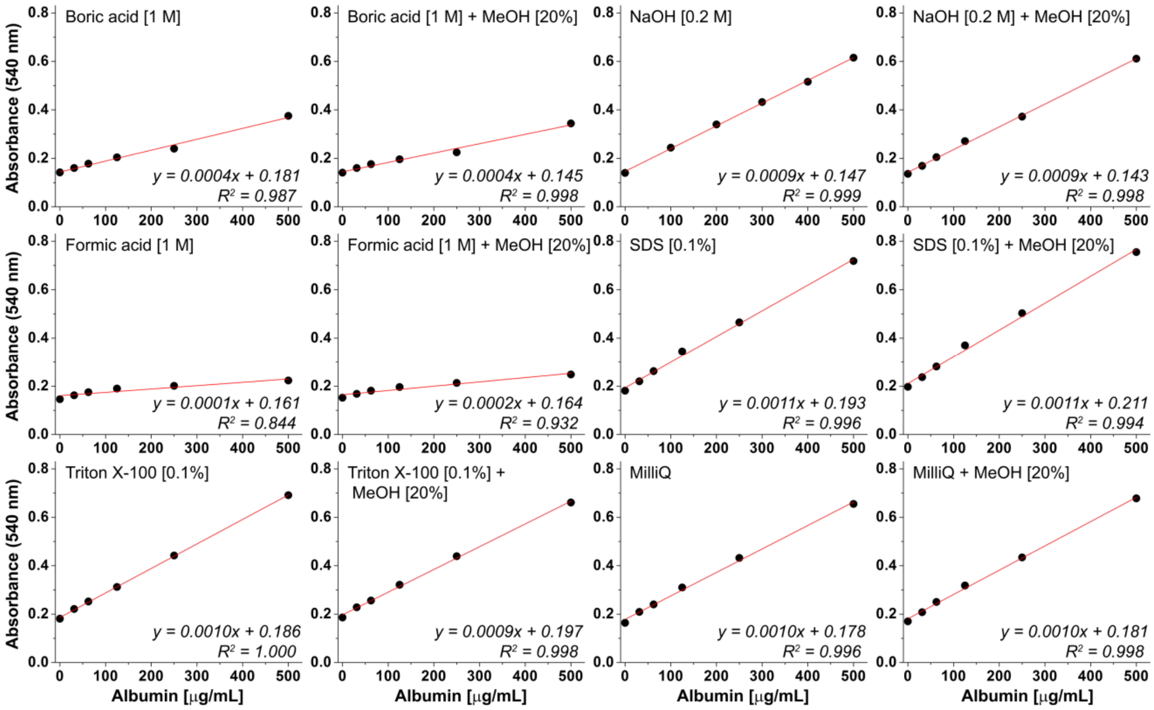

Figure S1. Protein concentration-absorbance plots for the BCA reaction using albumin as model protein and different cell lysis buffers, as indicated in the upper left corner. The linear fit function and goodness of fit are provided in the bottom right corner.

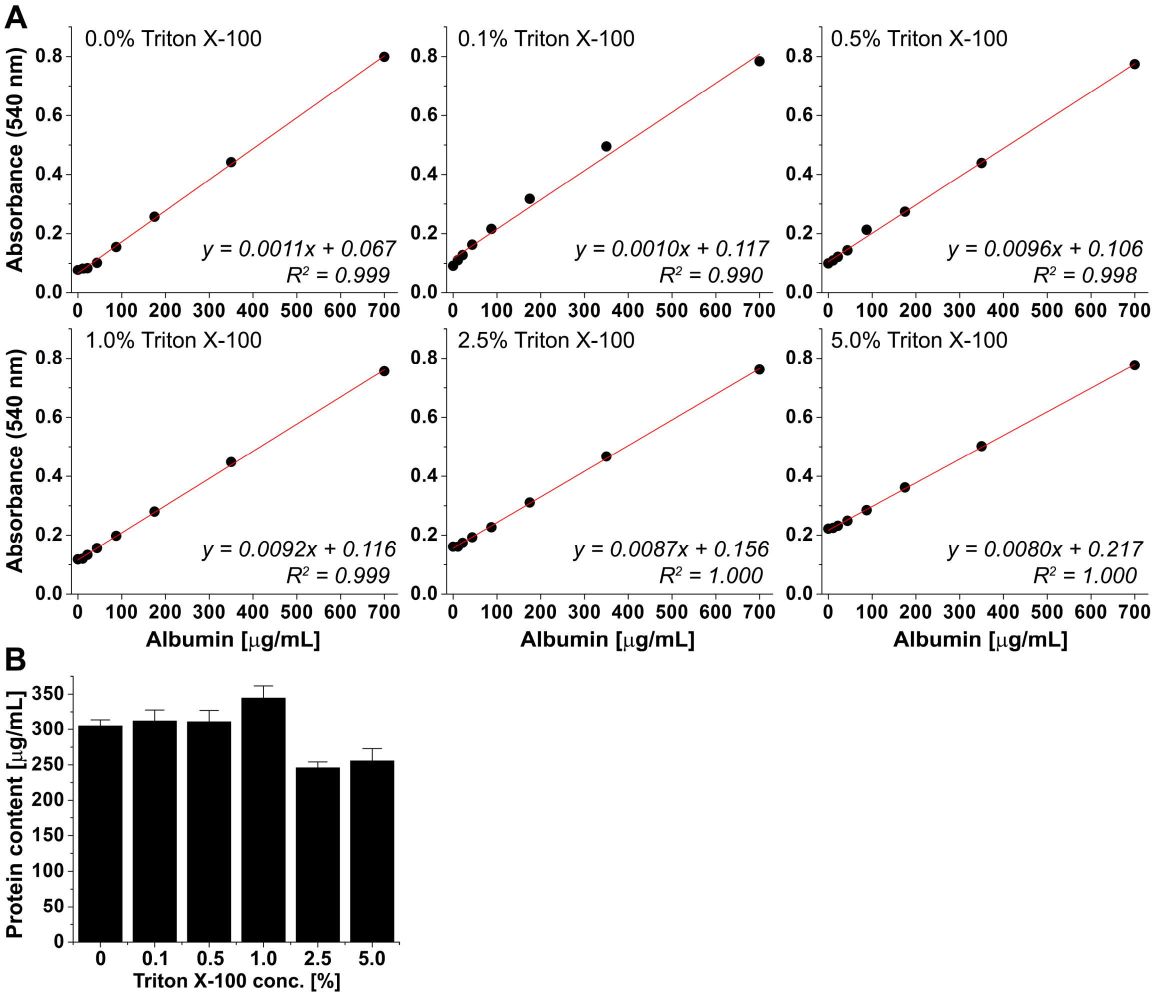

Figure S2. (**A**) Protein concentration-absorbance plots for the BCA reaction using albumin as model protein and increasing concentration of Triton X-100, as indicated in the upper left corner. The linear fit function and goodness of fit are provided in the bottom right corner. (**B**) Protein yield in cultured HepG2 cells following protein extraction using increasing concentrations of Triton X-100 in the cell lysis solution. Protein was determined with the BCA assay.

In conclusion, NaOH, MeOH, SDS, and Triton X-100 had no effect on the formation of the dimeric BCA chromophore. A Triton X-100 concentration of 1% in the cell lysis solution produced the greatest protein yield from cultured HepG2 cells. In line with these data, the cell lysis solution was composed of 0.1 M NaOH and 1% Triton X-100 for all protein determinations using BCA.

S2.11. Real-time analysis of oxidant formation during in vitro anoxia/reoxygenation in HepG2 cells

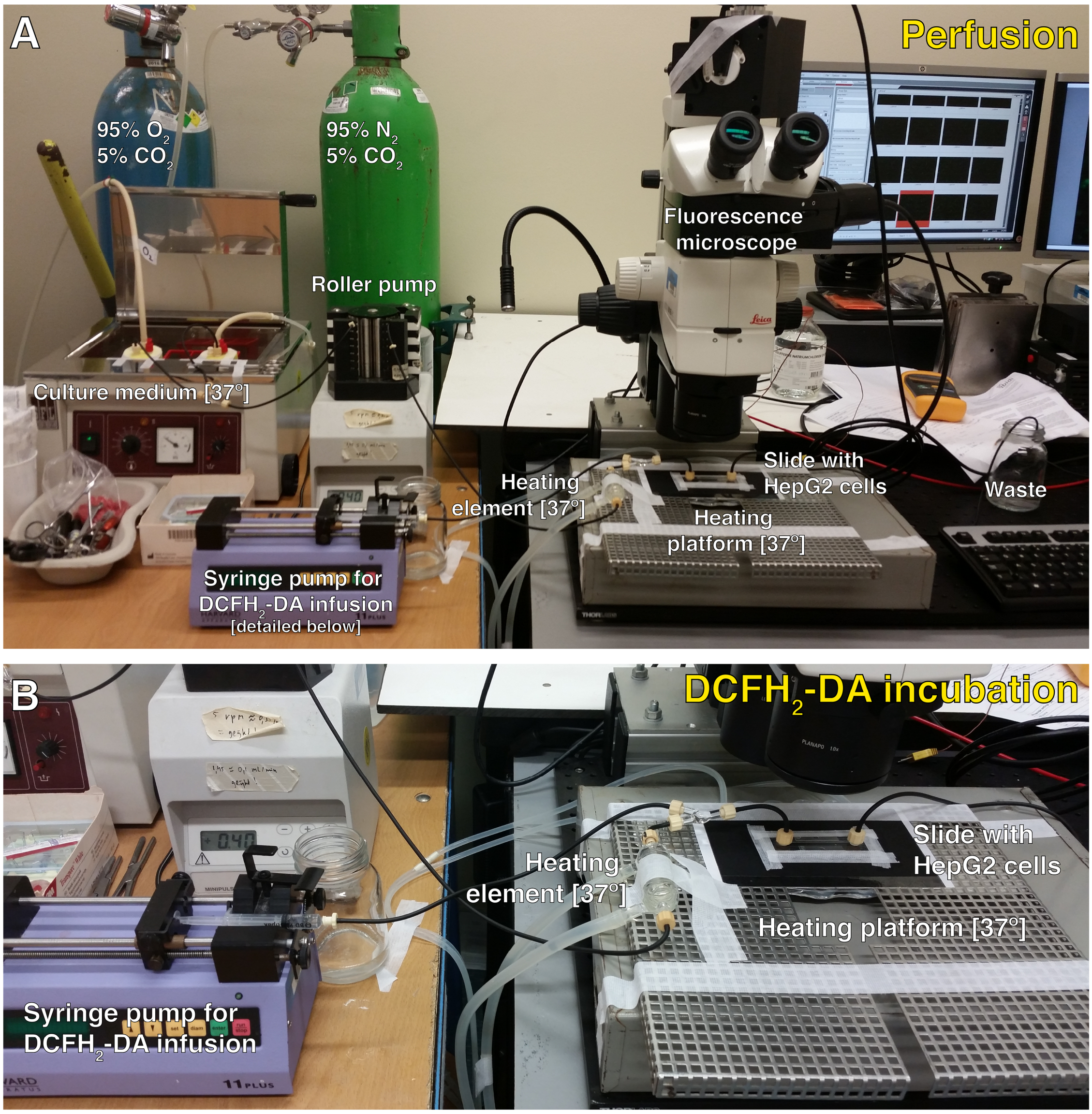

Figure S3. Perfusion setup for *in vitro* anoxia-reoxygenation experiments. Perfusion setup for the real-time analysis of oxidant formation during in vitro anoxia/reoxygenation in HepG2 cells. (**A**) During perfusion and (**B**) during incubation with $DCFH_2$-DA.

S3. Results

S3.1. The spectral properties of $DCFH_2$-DA and derivatives are pH-dependent

S3.1.1. Ground state absorption spectra of DCF, $DCFH_2$, and $DCFH_2$-DA

The spectral properties of $DCFH_2$-DA and its derivatives were determined with absorption and fluorescence spectroscopy. This is of particular interest when perfoming experiments that require the determination of probe concentrations and when studying DCF formation under conditions of varying pH. The molecular structures of DCF, $DCFH_2$, and $DCFH_2$-DA and the pKa values are shown in Figure 1 (main text). The concentration-dependent absorption spectra of DCF and its derivatives in different solvents are presented in Figure S4. The absorbance-concentration relationship was linear ($R^2 \geq 0.99$) for all compounds in the 0 - 20-μM concentration range. This, in combination with the fact that no blue or red bands appear in the main absorption band with increasing solute concentration, indicates that DCF and its derivatives do not form H-type and J-type aggregates (which would affect the fluorescence properties), respectively, at these experimentally relevant concentrations.

The main absorption band of oxidized DCF in water (Figure S4A) and HEPES buffer (Figure S4B) is structureless and exhibits a maximum (λ_{max}) at 503 nm that is attributable to π→π* transitions in the conjugated system. The blue shoulder at ~475 nm, which is unaffected by HEPES or ions (Na^+ and Cl^-), reflects a vibronic transition of the delocalized electrons. The type of solvent exerted a significant effect on the electronic and vibronic transitions, as polar H-bond donating and accepting solvent (MeOH) induced a bathochromic shift of the main absorption band (λ_{max} = 513 nm) and broadened the vibronic transition (Figure S4C). Polar H-bond accepting solvent (DMSO) exacerbated these effects (λ_{max} = 535 nm, Figure S4D), which was expected given the energy-lowering effect of polar solvents on π→π* transitions.

Protonation of the C_α, which yields reduced DCF ($DCFH_2$, Figure 1, main text), abrogates the electron delocalization over the fluorescein moiety as evidenced by the blue-shifted absorption band in all solvents (Figure S4E–H). Consequently, the $DCFH_2$ absorption spectrum adopts the spectral features of o-substituted benzoic acid [2]. The primary band in the deep UV region in all solvents corresponds to the π→π* transitions in the substituted benzenes and is sensitive to solvent and pH effects. The secondary band, which exhibits λ_{max} = 286 nm and 305 nm in the polar H-bond donating and accepting solvents, is attributable to an n→π* transition in the 2-chlorophenol moiety given the hypsochromic shift of this band when the hydroxyl group is substituted by acetate ($DCFH_2$-DA versus $DCFH_2$, Figure S4I–K vs. E–H). Additionally, in water (Figure S4E), HEPES buffer (pH = 6, Figure S4F), and MeOH (Figure S4H) the secondary band contains a red shoulder that can be ascribed to the additional valence electrons of the phenolate anion that forms at increasing basicity (pK_a = 9.2 [3], Figure 1, main text). The ionization results in lowering of the n→π* transition energy, as has been

shown for other substituted phenols such as tert-butylphenol, which undergoes a 15-nm bathochromic shift (275→290 nm) upon formation of the tert-butylphenoxide ion. Accordingly, the secondary band of $DCFH_2$ is red-shifted (λ_{max} = 305 nm) at pH >> 9.2 (Figure S4G), broadened, and more structureless and slightly hyperchromic. The gradual disappearance of the red shoulder in the secondary band of $DCFH_2$ with increasing pH has been reported by [4].

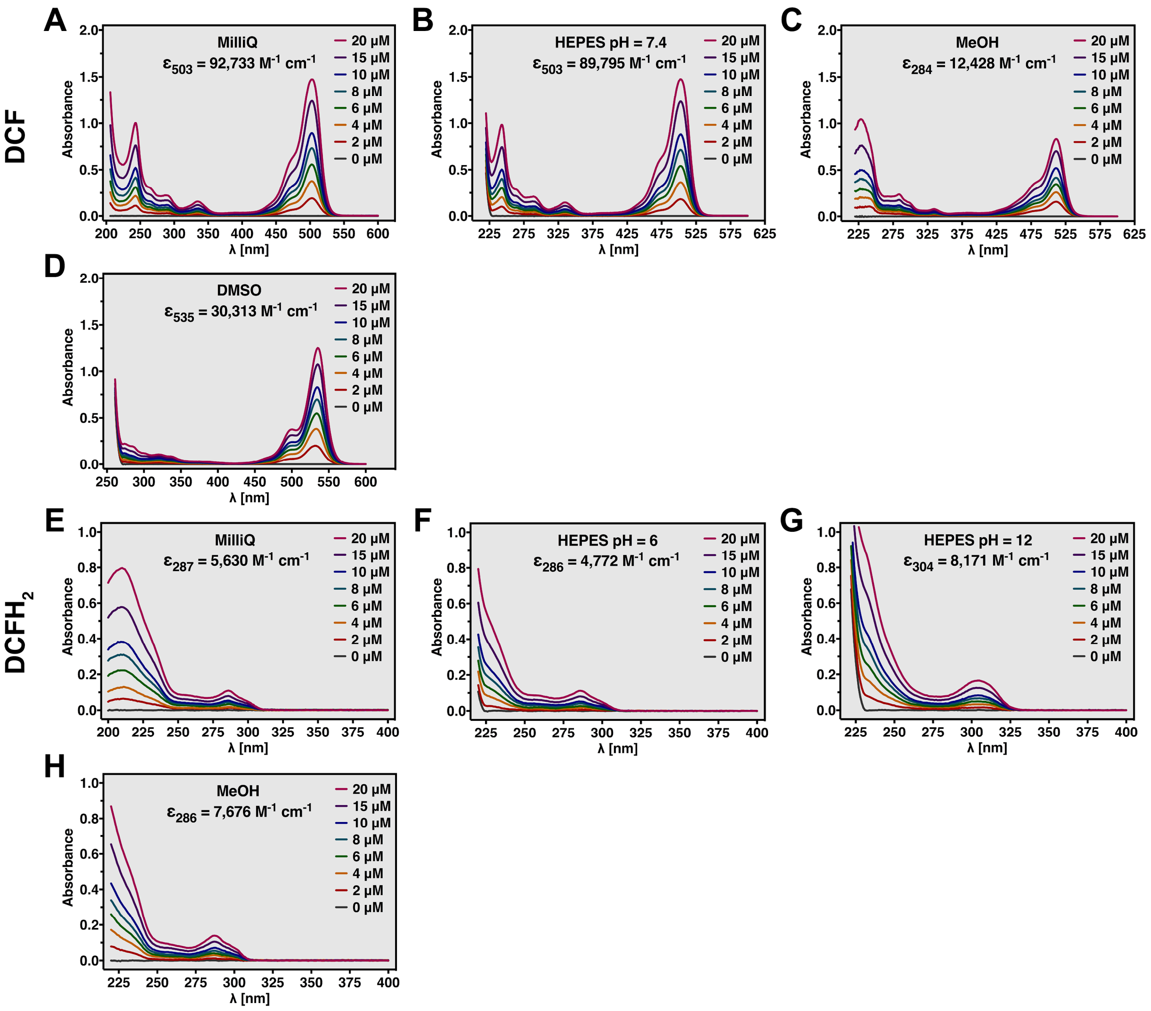

Figure S4. Absorption spectra and molar extinction coefficients of DCF and $DCFH_2$ in aqueous and organic solvent. Concentration-dependent absorption spectra (0 - 20 µM) and molar extinciton coefficients (ε) of (**A**) DCF in water (pH not adjusted), (**B**) HEPES buffer (pH = 7.4), (**C**) MeOH, and (**D**) DMSO. Concentration-dependent absorption spectra (0 - 20 µM) and molar extinciton coefficients (ε) of (**E**) $DCFH_2$ in water, HEPES buffer adjusted to (**F**) pH = 6 or (**G**) pH = 12, and (**H**) MeOH.

Acetylation of $DCFH_2$ at the phenolic hydroxyl groups (i.e., $DCFH_2$-DA) causes a hypsochromatic shift of both the primary and secondary absorption band (Figure S5). Inasmuch as the spectral properties of $DCFH_2$-DA are irrelevant for the practical applicability of this redox-sensitive fluorogenic probe beyond the determination of solute concentrations in different solvents (discussed below), the spectral properties of $DCFH_2$-DA will not be further discussed here.

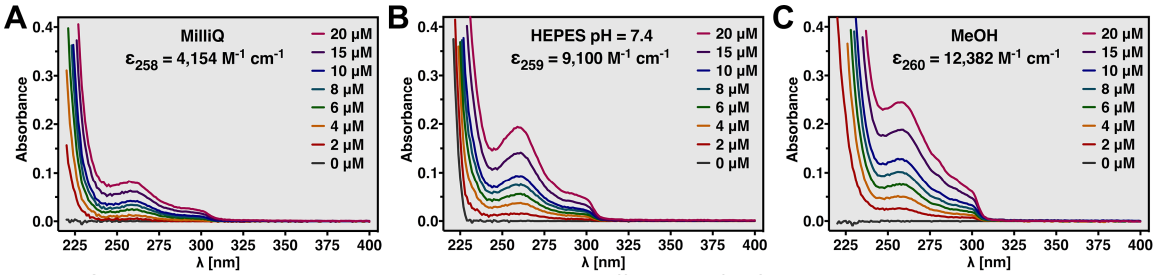

Figure S5. Absorption spectra and molar extinction coefficients of $DCFH_2$-DA in aqueous and organic solvent. Concentration-dependent absorption spectra (0 - 20 μM, λ = 220 - 600 nm) and molar extinction coefficients (ε) of (**A**) $DCFH_2$-DA in water, (**B**) HEPES buffer (pH = 7.4), and (**C**) MeOH.

S3.1.2. Molar extinction coefficients of DCF, $DCFH_2$, and $DCFH_2$-DA

The molar extinction coefficient of $DCFH_2$-DA and its derivatives was determined so as to enable the measurement of solute concentrations in the most relevant solvents in terms of solubility and in vitro application. The measured ε-values are presented in Figures S4 and S5. Moreover, the molar extinciton coefficient of $DCFH_2$ is useful for the simple yet accurate determination of the deacetylation efficacy and product yield following preparation from $DCFH_2$-DA. Given the high molar absorptivity of DCF, which translates to a lower limit of detection in absorbance-based methods relative to compounds with a lower molar extinciton coefficient, the molar extinciton coefficient of DCF can also be employed for the quantitation of intracellular DCF concentrations to determine cellular uptake or as a measure of oxidative stress.

S3.1.3. Changes in pH affect the ground state absorption spectrum of $DCFH_2$

With respect to practical applications, the effects of pH on the spectral properties of the chromophore and fluorophore can have considerable implications on the accuracy of the measurements. The pH of

the probe-based standard solution should therefore always be synchronized with the pH of the probe-containing sample when performing quantitative analysis. In Figure S4F and G it was shown that the ground state absorption spectrum of $DCFH_2$ in HEPES buffer was affected by solvent pH, which was also reported for $DCFH_2$ in PBS solution [4].

Figure S6A shows that pH-dependent effects on the absorption spectrum of $DCFH_2$ also apply in non-buffered aqueous solvent, but that these effects are mainly confined to the primary absoption band in the pH = 1 – 10 range. The $n \rightarrow \pi^*$ transition of the chlorophenol moiety that gives rise to the secondary absorption band is not markedly amenable to pH-related changes in this range. At pH < pK_a of the benzoic acid moiety (Figure 1, main text), the peak at 212 nm that is normally present at pH = 4 - 10 is relatively indiscriminate and the bands exhibit a red shoulder at 220 nm. Following deprotonation of the benzoic acid (pH ≥ 4), a well-defined peak appears at 212 nm that increases in intensity with pH, at least up to pH = 8 (i.e., second pK_a of $DCFH_2$). Also, the red 220-nm shoulder exhibits a bathochromic shift to 230 nm. At pH ≥ 11, where $DCFH_2$ is in fully deprotonated state, the primary peak undergoes a significant positional shift to 230 nm, indicative of lowering of the $\pi \rightarrow \pi^*$ transition energy in the substituted benzenes. More importantly, the secondary band undergoes a 18-nm bathochromic shift to 305 nm at pH ≥ 11, although the amplitude at 287 nm does not change considerably.

From a practical point of view, the secondary absorption band that peaks at 287 nm is most important for the determination of $DCFH_2$ concentration in solvent systems when using the Beer-Lambert law. The primary band is subject to much spectral interference from competing chromophores, including some buffer solutes. In the most important pH range, i.e., pH = 4 - 10, the secondary band can be used at 287 nm for spectrophotometric $DCFH_2$ assays without suffering from pH effects or other (chromatic) interferences. In biological or proteinaceous samples, however, spectrophotometric analysis/quantitation may be impossible due to the presence of amino acids with similar spectral properties (e.g., tryptophan and tyrosine residues [5]).

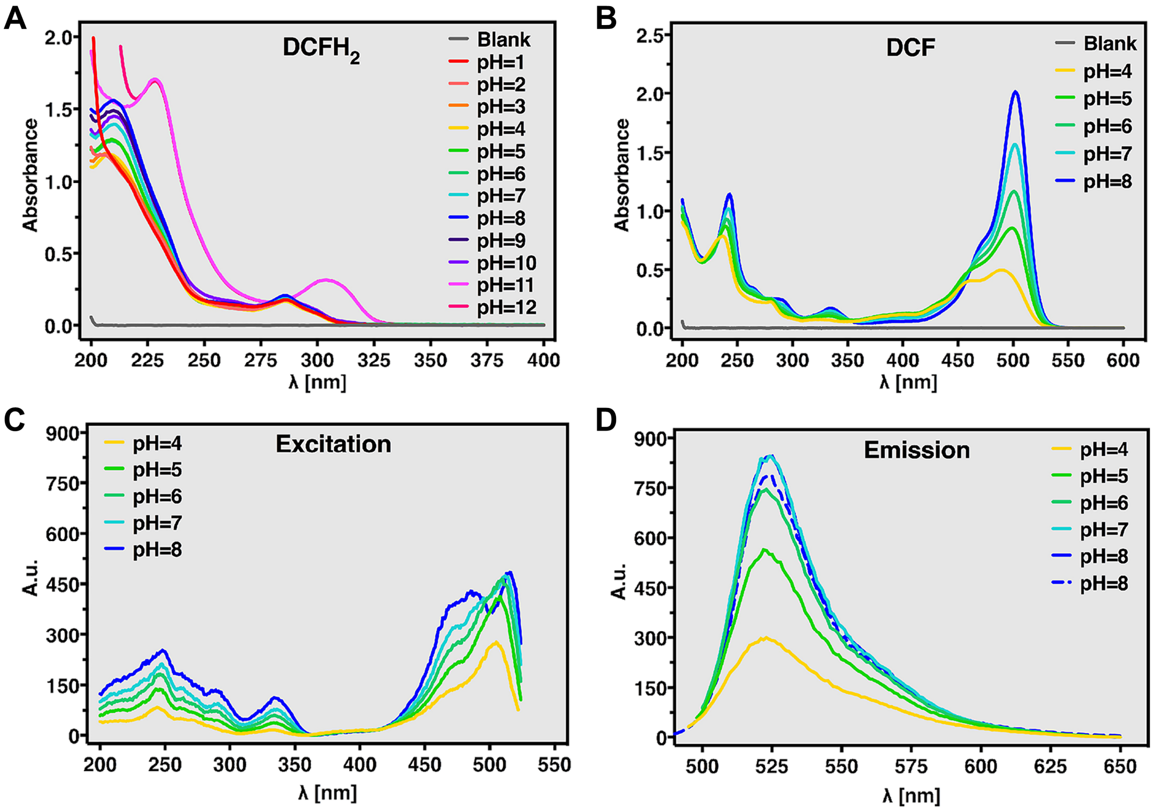

Figure S6. pH-dependent spectral properties of $DCFH_2$ and DCF in water. (**A**) pH-dependent absorption spectra (pH = 1 - 12, λ = 200 - 400 nm) of 20 µM $DCFH_2$ in water (adjusted with 37% HCl or 10 M NaOH). pH-dependent (pH = 4 - 8) (**B**) absorption (λ = 200 - 600 nm), (**C**) fluorescence excitation (λ = 200 - 525 nm at 545 nm emission), (**D**) and fluorescence emission spectra (λ = 525 - 650 nm at 480 nm excitation) of 20 µM DCF in water (adjusted with 37% HCl or 10 M NaOH).

S3.1.4. Changes in pH affect the absorption spectrum and fluorescence emission and excitation spectra of DCF

Unlike $DCFH_2$'s secondary band, the ground state absorption spectrum (Figure S6B) as well as fluorescence excitation (Figure S6C) and emission spectra (Figure S6D) of DCF were strongly affected by pH, in line with what has been reported previously [6]. With respect to absorption, the $\pi \rightarrow \pi^*$ transitions in the delocalized system, which give rise to the 502-nm peak at neutral pH, as well as the vibronic transitions of the delocalized electrons, which account for the blue shoulder at 475 nm, were altered by protonation of the second chlorophenol (pK_a = 7.9, Figure 1, main text) that in turn reduced the molar absorptivity at 503 nm. At pH = 5 and particularly at pH = 4 (i.e., the pK_a of the benzoic acid, Figure 1), the primary band underwent a hypsochromic shift of 13 nm and an additional

blue shoulder appeared at ~430 nm, likely reflecting vibronic transitions of electrons in the benzoic acid moiety.

Given that the fluorescence emission intensity is generally proportional to the molar extinction coefficient at equal excitation light intensity, wavelength, and fluorophore concentration, it was expected that DCF's fluorescence spectra would reflect the trend of the absorption spectra at different pH levels. In contrast, protonation of the chlorophenol (pH = 8 → 7) reduced the molar extinction coefficient (Figure S6B) but did not affect fluorescence emission intensity (Figure S6D), despite the lower excitation spectrum intensity at 480 nm at pH = 7 compared to pH = 8 (Figure S6C). The pronounced double peak in the excitation spectrum of the pH = 8 trace disappeared at pH < 8, indicating a shift in electronic transition states as a result of hydroxyl group protonation. The Stokes shift also became larger at increasing acidity, owing to the blue shifting of the excitation maximum. Moreover, the reduction in fluorescence intensity at pH = 5 and 4 was disproportional relative to that at pH = 6 (Figure S6D) and the reduction in molar absorbtivity (Figure S6B), suggesting that protonation of the benzoic acid reduced the quantum yield of DCF.

Consequently, the pH sensitivity that affects both DCF ground state absorption spectra and fluorescence spectra has a profound effect on the spectroscopic quantitation of oxidative stress and the formation of radical transients in various media and biological samples. The pH-dependent effects were deliberately measured at physiologically relevant alkaline (pH = 8) and acididic (pH = 4 - 6) conditions and juxtaposed to neutral pH. Alkaline and acidic pH levels are encountered during respiratory and metabolic alkalosis and acidosis (e.g., ischemia-reperfusion in the liver [7,8] and heart [9,10]), in cultured cancer cells and tumors *in vivo*, where intracellular pH is generally more acidic [11,12], and in organelles such as lysosomes [13]. Accordingly, the use of DCF for measuring oxidative stress or radical transient formation must account for the chemical milieu, especially since DCF is water-soluble and hence directly susceptible to shifts in pH.

Spectrally, the pH dependence should not be taken lightly because differential pH levels between experimental groups could skew or invalidate the interpretation of the DCF read outs. For example, when employing $DCFH_2$(-DA) in tumor xenografts in comparison to non-cancerous tissue, the fluorescence acquired from tumor tissue may yield an underestmation of oxidative stress or radical transient formation due to the aforementioned acidity in tumors, which would translate to reduced fluorescence (Figure S6D) at equal oxidation rate. These effects could also become manifest in cell cultures that vary in their pH, for instance due to differences in cell density, energy metabolism and/or

oxygenation. Similar principles apply to organs exposed to lactic acidosis, such as *in vivo* ischemia-reperfusion models for the heart [14] and liver [15,16]. In these models, the control group typically embodies an organ not subjected to ischemia-reperfusion. Due to ischemia-induced metabolic acidosis in the $DCFH_2$-laden cells, the ischemia-reperfusion group will likely yield undervalued levels of oxidative stress/radical transient formation.

S3.2. The stability of $DCFH_2$-DA and $DCFH_2$ in aqueous solvent and medium is dependent on the composition of the solution

$DCFH_2$ and $DCFH_2$-DA were previously shown to be stable in MeOH and DMSO for at least 28 d when stored at -20 °C [17]. Similar results were obtained for $DCFH_2$-DA stored in water or HEPES buffer at -20 °C (Figure S7). Significant DCF formation was observed when $DCFH_2$-DA was kept at 4 °C in both solvents, the onset of which was more rapid in HEPES buffer compared to water. This difference likely stems from more rapid $DCFH_2$-DA deacetylation at higher pH, i.e., pH = 7.4 for HEPES buffer versus pH ≈ 6 for water.

$DCFH_2$ proved less stable in aqueous solvent compared to $DCFH_2$-DA. Although $DCFH_2$ auto-oxidation in water was limited at -20 °C, it was considerably higher in samples stored at 4 °C (Figure S7A). In contrast, although $DCFH_2$ was less stable in HEPES buffer compared to water at -20 °C, a lesser extent of DCF formation was observed in samples dissolved in HEPES buffer at 4 °C after 28 d (Figure S7B). However, the latter observation was preceded by higher baseline DCF levels in HEPES buffer compared to water (0.164 ± 0.004% and 0.102 ± 0.015% of 20 μM DCF, respectively). These contradictory findings may stem from the complex interplay between the probe, HEPES, ionic strength, temperature, and pH. Although there is insufficient data to draw definitive conclusions about these effects, some observations exact close attention due to their practical relevance for the use of $DCFH_2$(-DA) *in vitro*.

First, the observed difference in $DCFH_2$ stability in HEPES buffer compared to water at -20 °C may be attributed to the reduction in freezing point that coincides with increasing osmolarity. Consequently, molecular interactions are hampered to a lesser extent in HEPES buffer, an effect that likely favors $DCFH_2$ auto-oxidation through enhanced formation of the DCFH semiquinone radical ($DCFH^{\bullet}/DCF^{\bullet-}$) [4] as well as its subsequent reaction with O_2 that results in the formation of DCF and $O_2^{\bullet-}$ [18]. Second, the difference in baseline DCF formation in samples kept at 4 °C most likely results

from transition metal traces in one or more buffer components [19]. Although the presence of trace amounts of transition metals has been described for HEPES [19], no such information was available for the HEPES-Na salt used in our experiments (Table S1). Third, the higher extent of overall $DCFH_2$ auto-oxidation in water compared to HEPES buffer at 4 °C presumably relates to the presence of buffer components. HEPES is reactive towards a variety of oxidants, including $O_2^{\bullet-}$ [20], H_2O_2 [21], $^{\bullet}OH$ [20], and Fe^{2+} [20]. More specifically, peroxynitrite can oxidize the piperazine moiety into an amino radical ($HEPES^{\bullet+}$/$HEPES^{\bullet}$) [22] through the generation of downstream radicals in the form of $^{\bullet}OH$, $^{\bullet}NO_2$, or $CO_3^{\bullet-}$ [23]. Like $DCFH^{\bullet}$, $HEPES^{\bullet}$ reacts with O_2 to form $O_2^{\bullet-}$ and $HEPES^{+}$, which can undergo additional oxidation [22,24]. $HEPES^{\bullet}$ generated in solutions containing $DCFH_2$ could therefore react with $DCFH^{\bullet}$ instead of O_2, thereby preventing the formation of DCF as well as $O_2^{\bullet-}$. However, the reactivity of $HEPES^{\bullet}$ towards O_2 ($k \approx 10^9$ M^{-1} s^{-1} [22]) renders it more likely that the excess of HEPES over $DCFH_2$ in this experiment (10 mM HEPES versus 20 μM $DCFH_2$) functions as an oxidant-scavenging sink that prevents radicals from reacting with $DCFH_2$.

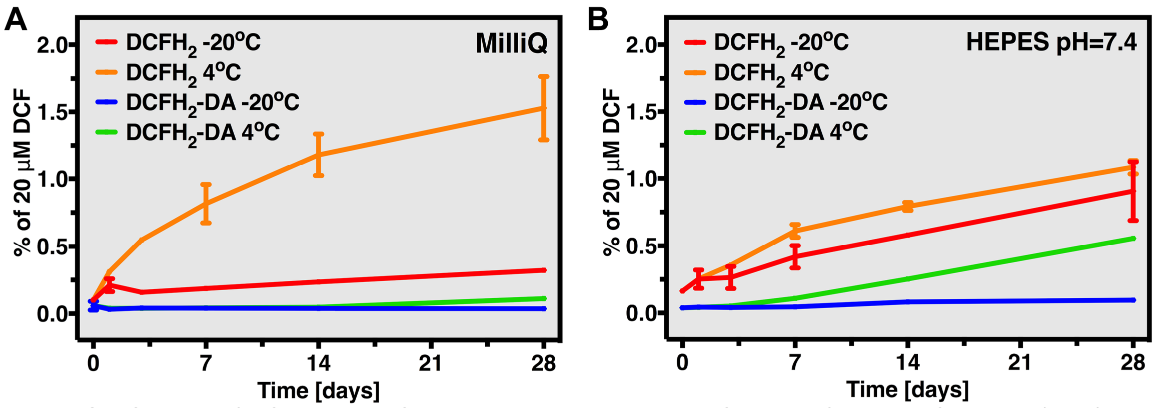

Figure S7. Stability of $DCFH_2$ and $DCFH_2$-DA in aqueous solvent. Stability of 20 μM $DCFH_2$-DA (blue/green line) and $DCFH_2$ (red/orange line) in (**A**) water and (**B**) HEPES buffer (10 mM HEPES, 0.88% NaCl, pH = 7.4, 0.292 osmol/kg) stored at -20 °C (red/blue line) or 4 °C (orange/green line) over a period of 28 d. $DCFH_2$-(DA) (auto-)oxidation was measured as DCF and plotted as a percentage of a freshly-prepared 20-μM DCF reference sample.

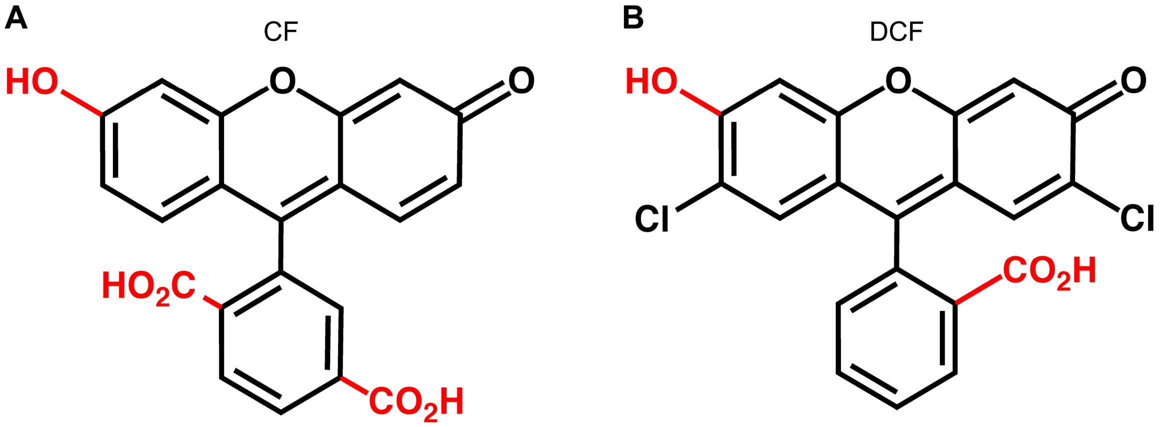

Figure S8. Chemical structure of (**A**) CF and (**B**) DCF.

References

1. Smith PK, Krohn RI, Hermanson GT, Mallia AK, Gartner FH, Provenzano MD, Fujimoto EK, Goeke NM, Olson BJ, Klenk DC (1985) Measurement of protein using bicinchoninic acid. Anal Biochem 150 (1):76-85. doi:10.1016/0003-2697(85)90442-7
2. Kamath BV, Mehta JD, Bafna SL (1975) Ultraviolet absorption spectra: Some substituted benzoic acids. J Chem Technol Biotechnol 25:743-751
3. Wrona M, Patel K, Wardman P (2005) Reactivity of 2',7'-dichlorodihydrofluorescein and dihydrorhodamine 123 and their oxidized forms toward carbonate, nitrogen dioxide, and hydroxyl radicals. Free Radic Biol Med 38 (2):262-270. doi:10.1016/j.freeradbiomed.2004.10.022
4. Wrona M, Wardman P (2006) Properties of the radical intermediate obtained on oxidation of 2',7'-dichlorodihydrofluorescein, a probe for oxidative stress. Free Radic Biol Med 41 (4):657-667. doi:10.1016/j.freeradbiomed.2006.05.006
5. Edelhoch H (1967) Spectroscopic determination of tryptophan and tyrosine in proteins. Biochemistry 6 (7):1948-1954. doi:10.1021/bi00859a010
6. Leonhardt H, Gordon L, Livingston R (1971) Acid-base equilibriums of fluorescein and 2', 7'-dichlorofluorescein in their ground and fluorescent states. J Phys Chem 75:245-249
7. Nishimura Y, Romer LH, Lemasters JJ (1998) Mitochondrial dysfunction and cytoskeletal disruption during chemical hypoxia to cultured rat hepatic sinusoidal endothelial cells: the pH paradox and cytoprotection by glucose, acidotic pH, and glycine. Hepatology 27 (4):1039-1049. doi:10.1002/hep.510270420
8. Heijnen BH, Elkhaloufi Y, Straatsburg IH, Van Gulik TM (2002) Influence of acidosis and hypoxia on liver ischemia and reperfusion injury in an in vivo rat model. J Appl Physiol (1985) 93 (1):319-323. doi:10.1152/japplphysiol.01112.2001
9. Garlick PB, Radda GK, Seeley PJ (1979) Studies of acidosis in the ischaemic heart by phosphorus nuclear magnetic resonance. Biochem J 184 (3):547-554. doi:10.1042/bj1840547
10. Pieper GM, Todd GL, Wu ST, Salhany JM, Clayton FC, Eliot RS (1980) Attenuation of myocardial acidosis by propranolol during ischaemic arrest and reperfusion: evidence with 31P nuclear magnetic resonance. Cardiovasc Res 14 (11):646-653. doi:10.1093/cvr/14.11.646
11. Raghunand N, Altbach MI, van Sluis R, Baggett B, Taylor CW, Bhujwalla ZM, Gillies RJ (1999) Plasmalemmal pH-gradients in drug-sensitive and drug-resistant MCF-7 human breast

carcinoma xenografts measured by 31P magnetic resonance spectroscopy. Biochem Pharmacol 57 (3):309-312. doi:10.1016/s0006-2952(98)00306-2
12. Griffiths JR (1991) Are cancer cells acidic? Br J Cancer 64 (3):425-427. doi:10.1038/bjc.1991.326
13. Mindell JA (2012) Lysosomal acidification mechanisms. Annu Rev Physiol 74:69-86. doi:10.1146/annurev-physiol-012110-142317
14. Oei GT, Heger M, van Golen RF, Alles LK, Flick M, van der Wal AC, van Gulik TM, Hollmann MW, Preckel B, Weber NC (2015) Reduction of cardiac cell death after helium postconditioning in rats: transcriptional analysis of cell death and survival pathways. Mol Med 20:516-526. doi:10.2119/molmed.2014.00057
15. Kloek JJ, Marechal X, Roelofsen J, Houtkooper RH, van Kuilenburg AB, Kulik W, Bezemer R, Neviere R, van Gulik TM, Heger M (2012) Cholestasis is associated with hepatic microvascular dysfunction and aberrant energy metabolism before and during ischemia-reperfusion. Antioxid Redox Signal 17 (8):1109-1123. doi:10.1089/ars.2011.4291
16. Olthof PB, van Golen RF, Meijer B, van Beek AA, Bennink RJ, Verheij J, van Gulik TM, Heger M (2017) Warm ischemia time-dependent variation in liver damage, inflammation, and function in hepatic ischemia/reperfusion injury. Biochim Biophys Acta Mol Basis Dis 1863 (2):375-385. doi:10.1016/j.bbadis.2016.10.022
17. Reiniers MJ, van Golen RF, Bonnet S, Broekgaarden M, van Gulik TM, Egmond MR, Heger M (2017) Preparation and Practical Applications of 2',7'-Dichlorodihydrofluorescein in Redox Assays. Anal Chem 89 (7):3853-3857. doi:10.1021/acs.analchem.7b00043
18. Rota C, Chignell CF, Mason RP (1999) Evidence for free radical formation during the oxidation of 2'-7'-dichlorofluorescin to the fluorescent dye 2'-7'-dichlorofluorescein by horseradish peroxidase: possible implications for oxidative stress measurements. Free Radic Biol Med 27 (7-8):873-881. doi:10.1016/s0891-5849(99)00137-9
19. Welch KD, Davis TZ, Aust SD (2002) Iron autoxidation and free radical generation: effects of buffers, ligands, and chelators. Arch Biochem Biophys 397 (2):360-369. doi:10.1006/abbi.2001.2694
20. Grady JK, Chasteen ND, Harris DC (1988) Radicals from "Good's" buffers. Anal Biochem 173 (1):111-115. doi:10.1016/0003-2697(88)90167-4
21. Zhao G, Chasteen ND (2006) Oxidation of Good's buffers by hydrogen peroxide. Anal Biochem 349 (2):262-267. doi:10.1016/j.ab.2005.10.005
22. Kirsch M, Lomonosova EE, Korth HG, Sustmann R, de Groot H (1998) Hydrogen peroxide formation by reaction of peroxynitrite with HEPES and related tertiary amines. Implications for a general mechanism. J Biol Chem 273 (21):12716-12724. doi:10.1074/jbc.273.21.12716
23. Radi R (1998) Peroxynitrite reactions and diffusion in biology. Chem Res Toxicol 11 (7):720-721. doi:10.1021/tx980096z
24. Keynes RG, Griffiths C, Garthwaite J (2003) Superoxide-dependent consumption of nitric oxide in biological media may confound in vitro experiments. Biochem J 369 (Pt 2):399-406. doi:10.1042/BJ20020933
